# Hypoglycemic activity of *Garcinia mangostana* L. extracts on diabetes rodent models: A systematic review and network meta-analysis

**DOI:** 10.3389/fphar.2024.1472419

**Published:** 2024-10-02

**Authors:** Moragot Chatatikun, Aman Tedasen, Phichayut Phinyo, Pakpoom Wongyikul, Wiyada Kwanhian Klangbud, Fumitaka Kawakami, Motoki Imai, Sirithip Chuaijit, Sarawut Rachmuangfang, Siriporn Phuwarinyodsakul, Rattana Leelawattana, Atthaphong Phongphithakchai

**Affiliations:** ^1^ Department of Medical Technology, School of Allied Health Sciences, Walailak University, Nakhon Si Thammarat, Thailand; ^2^ Center of Excellence Research for Melioidosis and Microorganisms (CERMM), Walailak University, Nakhon Si Thammarat, Thailand; ^3^ Research Excellence Center for Innovation and Health Products (RECIHP), Walailak University, Nakhon Si Thammarat, Thailand; ^4^ Center for Clinical Epidemiology and Clinical Statistics, Department of Biomedical Informatics and Clinical Epidemiology (BioCE), Faculty of Medicine, Chiang Mai University, Chiang Mai, Thailand; ^5^ Medical Technology program, Faculty of Science, Nakhon Phanom University, Nakhon Phanom, Thailand; ^6^ Research Facility of Regenerative Medicine and Cell Design, School of Allied Health Sciences, Kitasato University, Sagamihara, Japan; ^7^ Department of Regulation Biochemistry, Kitasato University Graduate School of Medical Sciences, Sagamihara, Japan; ^8^ Department of Molecular Diagnostics, School of Allied Health Sciences, Kitasato University, Sagamihara, Japan; ^9^ Department of Medical Science, School of Medicine, Walailak University, Nakhon Si Thammarat, Thailand; ^10^ Endocrinology and Metabolism Unit, Division of Internal Medicine, Faculty of Medicine, Prince of Songkla University, Songkhla, Thailand; ^11^ Nephrology Unit, Division of Internal Medicine, Faculty of Medicine, Prince of Songkla University, Songkhla, Thailand

**Keywords:** *Garcinia mangostana*, mangosteen, diabetes mellitus, glucose, network meta-analysis

## Abstract

**Background:**

Diabetes mellitus is a significant global health issue, and alternative treatments from natural products like *Garcinia mangostana* L. [Clusiaceae] or GM are being explored for their potential benefits. This study focused on evaluating the hypoglycemic effects of GM on diabetic rodent models.

**Methods:**

A comprehensive search was conducted in PubMed, Scopus, and Embase for studies reporting blood glucose levels within 2 weeks as the primary outcome and changes in total cholesterol (TC), triglycerides (TG), low-density lipoprotein cholesterol (LDL-C), and high-density lipoprotein cholesterol (HDL-C) as secondary outcomes. A network meta-analysis (NMA) was performed to determine the pooled effectiveness of each intervention, estimating the weighted mean difference (WMD) and 95% confidence interval (CI) from both direct and indirect evidence. The surface under the cumulative ranking curve (SURCA) was used to rank the interventions.

**Results:**

Ten articles were identified, with nine included for quantitative analysis. All GM extracts showed greater effectiveness than the control in decreasing blood glucose levels within 2 weeks. GM at 200 mg/kg (GM200) was the top-ranked extract for reducing glucose levels beyond 2 weeks and increasing HDL-C levels. The ethanol extract of GM at 200 mg/kg (GME200) was the most effective for blood glucose reduction within 2 weeks and for TC and TG reductions. The methanol extract of GM at 200 mg/kg (GMM200) was the top-ranked extract for LDL-C reductions.

**Conclusion:**

GM and its extracts demonstrated significant hypoglycemic activity and improvements in lipid profiles in diabetic rodent models, highlighting their potential as therapeutic agents for the prevention and treatment of diabetes mellitus. Further research in human trials is warranted to confirm these findings and establish clinical applications.

**Clinical trial registration:**

https://www.crd.york.ac.uk/prospero/display_record.php?ID=CRD42023426254.

## 1 Introduction

Diabetes mellitus (DM) is a metabolic syndrome and endocrinopathy. Type 2 DM is the most common among all types of diabetes. DM is a chronic condition characterized by high blood glucose levels. Hyperglycemia or high blood sugar is an effect of uncontrolled diabetes mellitus that leads to damage in many organs (retinopathy, neuropathy, nephropathy, blood vessels, and cardiac problems) and activates oxidative stress in cell membranes by producing reactive oxygen species (Daryabor et al., 2020). The number of DM patients has rapidly increased in recent years and is predicted to reach about 643 million people by 2030 (Kumar et al., 2023). Metformin is an oral hypoglycemic drug, which is the first-line treatment for type 2 DM. However, metformin causes adverse effects including nausea, vomiting, diarrhea, abdominal disturbance, loss of appetite, and lactic acidosis (Shurrab and Arafa, 2020). Therefore, new agents from natural products may serve as alternative pharmacologic treatments due to their safety, effectiveness, and cost advantage over newer drugs (Rodríguez et al., 2022).

*Garcinia mangostana* L. [Clusiaceae] or GM is commonly known as mangosteen or “Queen of Tropical Fruits.” It is a tree cultivated in the tropical rainforest. Its fruit is dark purple or reddish with white, sweet, and juicy flesh. The pericarp (rind) of the GM fruit has been traditionally used by Southeast Asians for its anti-microbial properties in treating skin infections, wounds, and dysentery (Pedraza-Chaverri et al., 2008). In Ayurvedic medicine, the GM pericarp is widely utilized in India. Its hull contains α-mangostin, which has been traditionally used for treating inflammation, diarrhea, cholera, and dysentery (Bi et al., 2023). Today, GM fruit extract is widely commercialized as a functional food or drink, containing minor components such as vitamins, and is promoted for general health benefits, including its anti-inflammatory and weight loss properties (Udani et al., 2009; Xie et al., 2015). Mangosteen has been shown to contain a variety of secondary metabolites such as xanthones, isoflavones, tannins, flavonoids, procyanidin, benzophenones, and mangostin (Songsiriritthigul et al., 2020; Tran et al., 2021; Zheng et al., 2021). Xanthones have been isolated from the pericarp, whole fruit, barks, and leaves of mangosteen (Pedraza-Chaverri et al., 2008). Previous studies have shown that GM extracts have various biological effects including antioxidant, anti-allergic, anti-microbial, anti-tumoral, anti-inflammatory, anti-viral, and hypoglycemic activities (Pedraza-Chaverri et al., 2008; Cunha et al., 2014; Suthammarak et al., 2016; Primiani and Lestari, 2019; Tatiya-Aphiradee et al., 2019; Tarasuk et al., 2022).

Nowadays, the consumption of botanical drugs for complementary and alternative medicine has rapidly increased. An earlier review article confirmed the anti-diabetic potential of GM extracts (Yani et al., 2021). However, no systematic review or network meta-analysis (NMA) has been conducted to explore the effects of GM extracts on diabetes. Meta-analyses combine eligible published data to synthesize the results of studies on an intervention, providing more reliable and robust conclusions than a single study (Stone and Rosopa, 2017). Whereas pairwise meta-analyses compare the effectiveness of two treatments that have been directly compared in head-to-head trials, an NMA allows for the combination of both direct and indirect evidence, facilitating comparisons across a network of treatments (Rouse et al., 2017). In this study, we aimed to perform an NMA to evaluate and compare the hypoglycemic activity of GM extracts.

## 2 Methods

This systematic review (SR) and NMA adhered to the guidelines outlined in the Cochrane Handbook for Systematic Reviews of Interventions version 6.0, and its presentation follows the Preferred Reporting Items for Systematic Reviews and Meta-analyses (PRISMA) guidelines (Tricco et al., 2018; Higgins et al., 2022). The review protocol was registered in the PROSPERO database with the registration number CRD42023426254. PRISMA NMA checklist is shown in Supplementary Table S1.

### 2.1 Data sources and search strategy

To identify relevant research articles, a thorough search was conducted across the PubMed, Scopus, and Embase databases. The search period spanned from the inception of each database until 15 May 2023. The language was restricted to English. The search terms employed were as follows: (*G. mangostana* OR *G. mangostana* OR mangosteen OR *G. mangostana* extract OR *G. mangostana* extract OR mangosteen extract) AND (diabetes mellitus OR glucose OR hyperglycemia) (Supplementary Tables S2-S4).

### 2.2 Study selection and outcomes

Two authors, namely, M.C. and A.P., independently screened the data, meticulously evaluating the titles and abstracts of the retrieved research articles to determine the final list of included studies. In cases of any discrepancies or disagreements between the two research workers, a consensus was reached through discussions involving a third author, S.S.

The inclusion criteria of this study were defined as follows: 1) studies that reported the hypoglycemic activity of GM extract in rodent models with diabetes mellitus; 2) studies that used GM extract, regardless of the extracted part, the extraction methods, and administration routes; 3) studies that compared individual experiment studies with a diabetic control group using vehicles/placebo or standard treatment; 4) studies that reported the outcomes of interest; and 5) studies that were published in English. Exclusion criteria encompassed *in vitro* studies, isolated metabolites from GM, modified GM metabolites, or combination treatments with other botanical drugs or chemicals. Additionally, studies without separate control groups, those not published in English language, and those not reporting relevant outcome measures (especially blood glucose levels) were excluded.

The primary outcome measured at the endpoint within a 2-week period was the blood glucose level. Secondary outcomes included blood glucose levels beyond 2 weeks, along with measurements of total cholesterol (TC), triglycerides (TG), low-density lipoprotein cholesterol (LDL-C), and high-density lipoprotein cholesterol (HDL-C) in rodent models with diabetes mellitus and their relevant controls. All time periods for secondary outcomes were taken into consideration.

### 2.3 Data extraction and risk of bias assessment

After removing duplicates, preliminary screening was performed by reading the abstracts and titles of the remaining studies. According to the inclusion and exclusion criteria, the following data were extracted from each article by two authors (M.C. and A.P.): study design characteristics, utilized plant part, solvent employed, type of rodent DM models, sex, diet, details of interventions and control groups, duration of exposure to GM, route of administration, and predefined outcomes as described above. A third reviewer (S.S.) cross-checked the extracted data.

For continuous outcome parameters, the mean and standard deviation (SD) of the outcomes, along with the number of DM models, were extracted. For studies reporting the standard errors of means (SEM), the corresponding SDs were computed by multiplying the SEM by the square root of the respective sample size. The imputation of SDs was carried out according to the recommendations of the Cochrane Handbook for Systematic Reviews of Interventions (Higgins et al., 2022).

Authors of studies with missing or incomplete data were contacted by email to obtain additional information. If the corresponding author did not reply within a 2-week period, the request was repeated. If there was no response after the second attempt, the data were reported as not accessible and subsequently excluded from the analysis. The risk of bias for each individual study was assessed by three authors (M.C., A.P., and S.S.) in parallel using the RoB2: a revised Cochrane risk-of-bias tool for randomized trials.

### 2.4 Data synthesis and analysis

The NMA was performed using random effects to estimate the weighted mean difference (WMD) along with 95% confidence intervals (CI), which were calculated for all continuous outcome measures to compare the effectiveness of the intervention based on both direct and indirect evidence using Stata 17 (StataCorp, TX, United States) (Rücker et al., 2020). A *P*-value of less than 0.05 was considered statistically significant. To ensure the validity of the NMA estimates, we performed the consistency assumption using a global test, the loop-specific approach, and the node-splitting approach (Tonin et al., 2017; Watt et al., 2019). Additionally, we estimated the ranking probabilities for all treatments, indicating their probability of being at each possible rank for each treatment. Intervention ranking was based on the surface under the cumulative ranking curve (SURCA) (Christofilos et al., 2022). The intervention effects in head-to-head comparisons were presented in league tables, showing the WMD and 95% CI. Sensitivity analysis was employed to assess the robustness of the outcome estimates, primarily by systematically removing one study at a time and subsequently repeating the primary meta-analyses (Lin et al., 2016).

### 2.5 Grading the strength of evidence

Two reviewers (M.C. and A.P.) independently evaluated the strength of the evidence using the Confidence in Network Meta-Analysis (CINeMA) approach. This approach assesses six domains, including within-study risk of bias, reporting bias, indirectness, imprecision, heterogeneity, and inconsistency. The confidence in the estimated treatment effect derived from the NMA was summarized into four levels of evidence certainty: high, moderate, low, and very low.

## 3 Results

### 3.1 Search results

**FIGURE 1:**
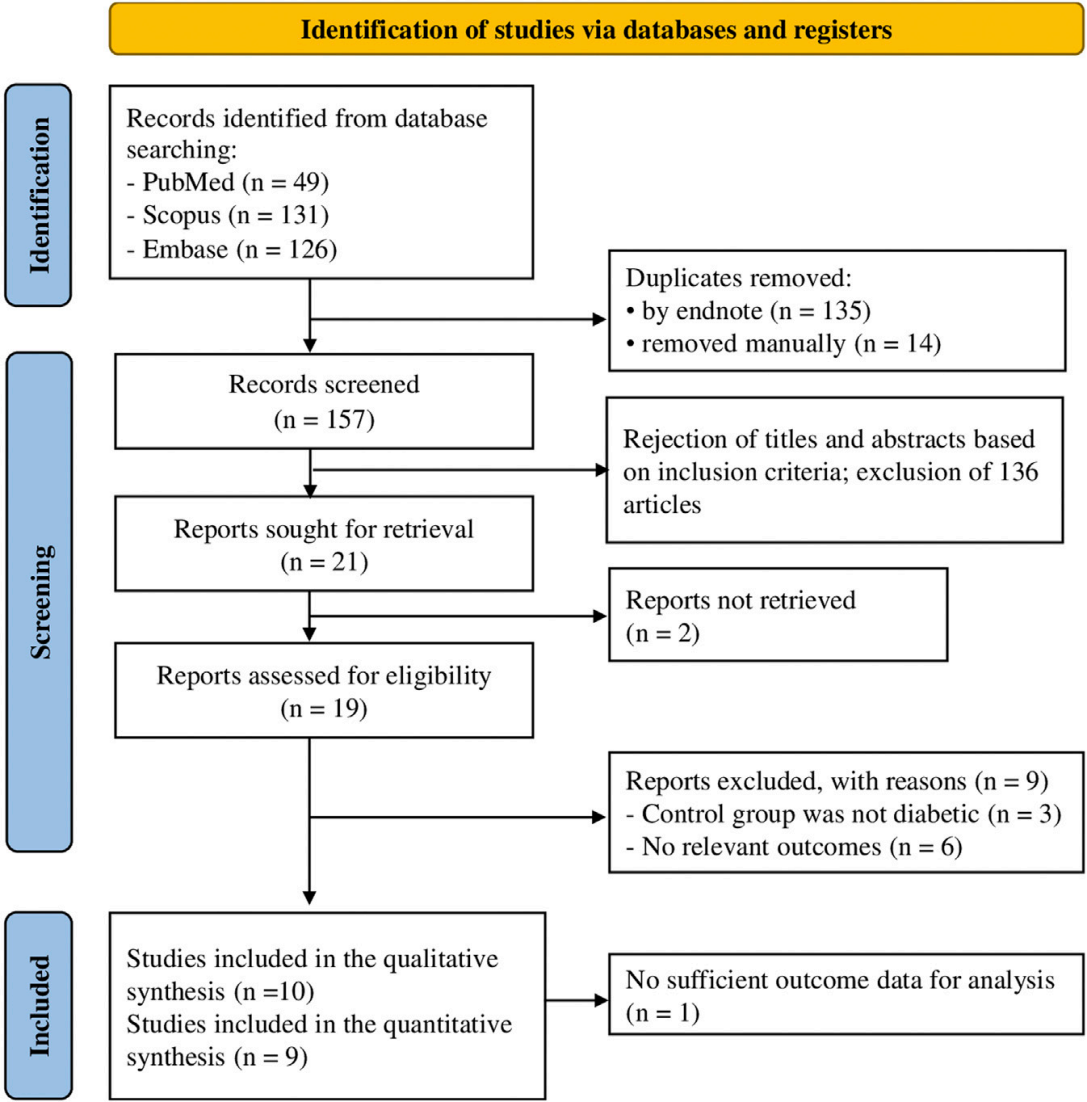
Selection process of the articles according to the Preferred Reporting Items for Systematic Reviews and Meta Analyses (PRISMA guideline).

The systematic literature search, as depicted in Figure 1, yielded a comprehensive collection of 306 articles, from three databases such as PubMed (49 articles), Scopus (131 articles), and Embase (126 articles). Before screening the remaining titles and abstracts, 149 articles were eliminated through the use of citation manager (135 articles) and manual review (14 articles). After removing the remaining titles and abstracts, 21 full texts of relevant studies were obtained. However, the majority of these studies were excluded from the original meta-analysis due to their failure to report diabetic control groups and blood glucose levels. Ultimately, 10 articles were eligible for inclusion in the quantitative synthesis, as depicted in Supplementary Figure S1.

### 3.2 Study characteristics

The characteristics of the 10 included studies are shown in Table 1. These studies were published from 2016 onward. Among the included studies, animals were treated with GM extracts isolated from peels in one study (10%), pericarps in seven studies (70%), and rinds in two studies (20%). The GM extracts were prepared using four different methods: maceration (60%), freeze-drying with dry ice and technical ethanol (10%), oven drying (10), and unspecified method (10%). GM extracted were then extracted with ethanol in five studies, hexane in one study, chloroform in one study, methanol in one study, and water in one study, whereas two studies did not provide data on the solvents used. Moreover, there was a study that bought the commercial product. The included studies utilized C57BL/6 mice in one study (10%), BALB/c mice in two studies (20%), ICR mice in two studies (20%), Wistar rats in three studies (30%), *Rattus norvegicus* in one study (10%), and Sprague–Dawley rats in one study (10%). To establish a diabetic model, two studies used animals induced by high-fat diet (HFD) (20%), two studies used animals induced by streptozotocin (STZ) (20%), and four studies used animals induced by both high-fat diet and STZ (40%). The types of drugs employed in the studies included orlistat (OLST), metformin (MET), and glibenclamide (GBC), with one study utilizing OLST (10%), one study utilizing MET (10%), and four studies utilizing GBC (40%). GM doses varied from 18 to 400 mg/kg body weight. For the primary outcomes in six studies, the blood glucose (BG) levels were selected within 2 weeks for the NMA. The blood glucose levels at more than 2 weeks in four studies were chosen for the secondary outcomes. Additionally, TC, TG, LDL-C, and HDL-C from four studies were chosen at any time for the NMA. The characteristics of studies are summarized in Table 1 (Chae et al., 2016; Taher et al., 2016; Husen et al., 2017; Karim et al., 2017; Husen et al., 2019; Karim et al., 2019; Primiani and Lestari, 2019; Sunarjo, 2020; Labban et al., 2021; Muniroh et al., 2021).

**TABLE 1:** Study characteristics.

Study ID	First author, year	Part	Extraction method	Solvent	Animal model	Sex	Diet	Intervention (n), dose (mg/kg MW)	Duration exposed to GM	Route administration	Outcome measures
1	Chae et al. (2016)	Peel	Maceration	Ethanol	C57BL/6 mice	Males	HFD	CTRL (8)	45 days	Oral	BG, TC, TG, HDL-C, LDL-C
								GME 50 mg/kg (8)			
								GME 100 mg/kg (8)			
								OLST 20 mg/kg (8)			
2	Husen et al. (2017)	Pericarp	Maceration	Ethanol	Mice of strain BALB/c, STZ	Males	HFD	CTRL (6)	14 days	Oral	BG
								GME 50 mg/kg (6)			
								GME 100 mg/kg (6)			
								GME 200 mg/kg (6)			
								MTF 100 mg/kg (6)			
3	Husen et al. (2019)	Pericarp	Freeze-drying (dry ice, technical ethanol), non-polar (hexane), semi-polar (chloroform), and polar (ethanol) extraction	- Non-polar (hexane) - Semi-polar (chloroform) - Polar (ethanol)	Mice of strain BALB/c, STZ	Males	Control	CTRL (6)	14 days	Oral	BG
								GMH 18 mg/kg (6)			
								GMC 80 mg/kg (6)			
								GME 50 mg/kg (6)			
4	Karim et al. (2017)	Rind	No data (commercial product)	No data (commercial product)	ICR mice, STZ	Males	HFD	CTRL (6)	7 days	Oral	BG, TC, TG, HDL-C, LDL-C
								GM 100 mg/kg (6)			
								GM 200 mg/kg (6)			
								GBC 60 mg/kg			
5	Karim et al. (2019)	Pericarp	Hot water extraction	Water	ICR mice, STZ	NR	HFD	CTRL (6)	7 days	Oral	BG
								GMA 100 mg/kg (6)			
								GMA 200 mg/kg (6)			
								GBC 60 mg/kg			
6	Labban et al. (2021)	Pericarp	Maceration	Methanol	Albino Wistar rats	Male	HFD	CTRL (5)	6 weeks	Oral	BG, TC, TG, HDL-C, LDL-C
								GMM 400 mg/kg (5)			
7	Muniroh et al. (2021)	Pericarp	Maceration	Ethanol	Wistar rats, STZ	Males	HFD	CTRL (6)	8 weeks	Oral	BG
								GME 100 mg/kg (6)			
								GME 200 mg/kg (6)			
								GME 400 mg/kg (6)			
8	Primiani and Lestari (2019)	Pericarp	Oven drying	NR	Rats (*Rattus norvegicus*), STZ	Male	Normal	CTRL (6)	36 days (no SD)	Oral	BG
								GM 83.3 mg/kg (6)			
								GBC 0.09 mg/kg (6)			
9	Sunarjo (2020)	Rind	Maceration	NR	Wistar rats, STZ	Male	Normal	CTRL (10)	10,14 days (no SD)	Oral	BG
								GM 100 mg/kg (10)			
10	Taher et al. (2016)	Pericarp	Maceration	Ethanol	Sprague–Dawley rats, STZ	Male	Normal	CTRL (6)	7, 14, 21, 28 days	Oral	BG, TC, TG, HDL-C, LDL-C
								GME 50 mg/kg (6)			
								GME 100 mg/kg (6)			
								GME 200 mg/kg (6)			
								GBC 0.5 mg/kg (6)			

Note: BG, blood glucose; CTRL, control; GBC, glibenclamide; GM, Garcinia mangostana extract; GMA, aqueous extract of *Garcinia mangostana*; GMC, chloroform extract of *Garcinia mangostana*; GME, ethanol extract of *Garcinia mangostana*; GMH, hexane extract of *Garcinia mangostana*; GMM, methanol extract of *Garcinia mangostana*; HDL-C, high-density lipoprotein cholesterol; LDL-C, low-density lipoprotein cholesterol; MTF, metformin; NR, not reported; OST, orlistat; STZ, streptozotocin; TC, total cholesterol; TG, triglyceride.

### 3.3 Risk of bias

**FIGURE 2:**
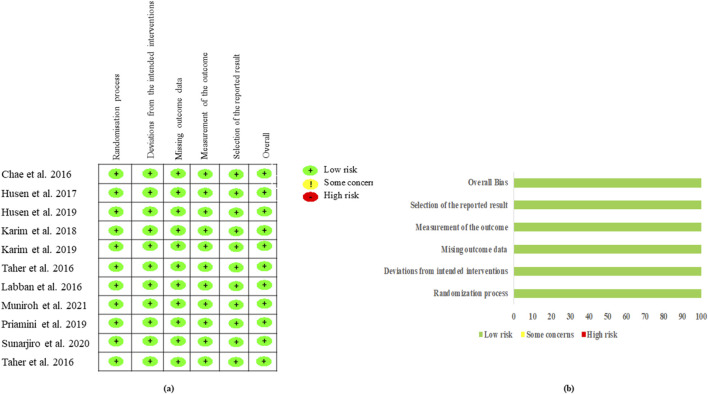
Risk of bias of the included randomized studies evaluated by the Cochrane RoB2 tool. **(A)** Risk of bias summary: review authors’ judgments about each risk of bias item for each included study and **(B)** risk of bias graph: review authors’ judgments about each risk of bias item presented as percentages across all included studies.

The results of the reporting bias assessment are shown in Figure 2. Regarding the quality of the studies, all 10 studies were regarded as having a low risk of bias based on the ROB2 tool.

### 3.4 Evidence of network

**FIGURE 3:**
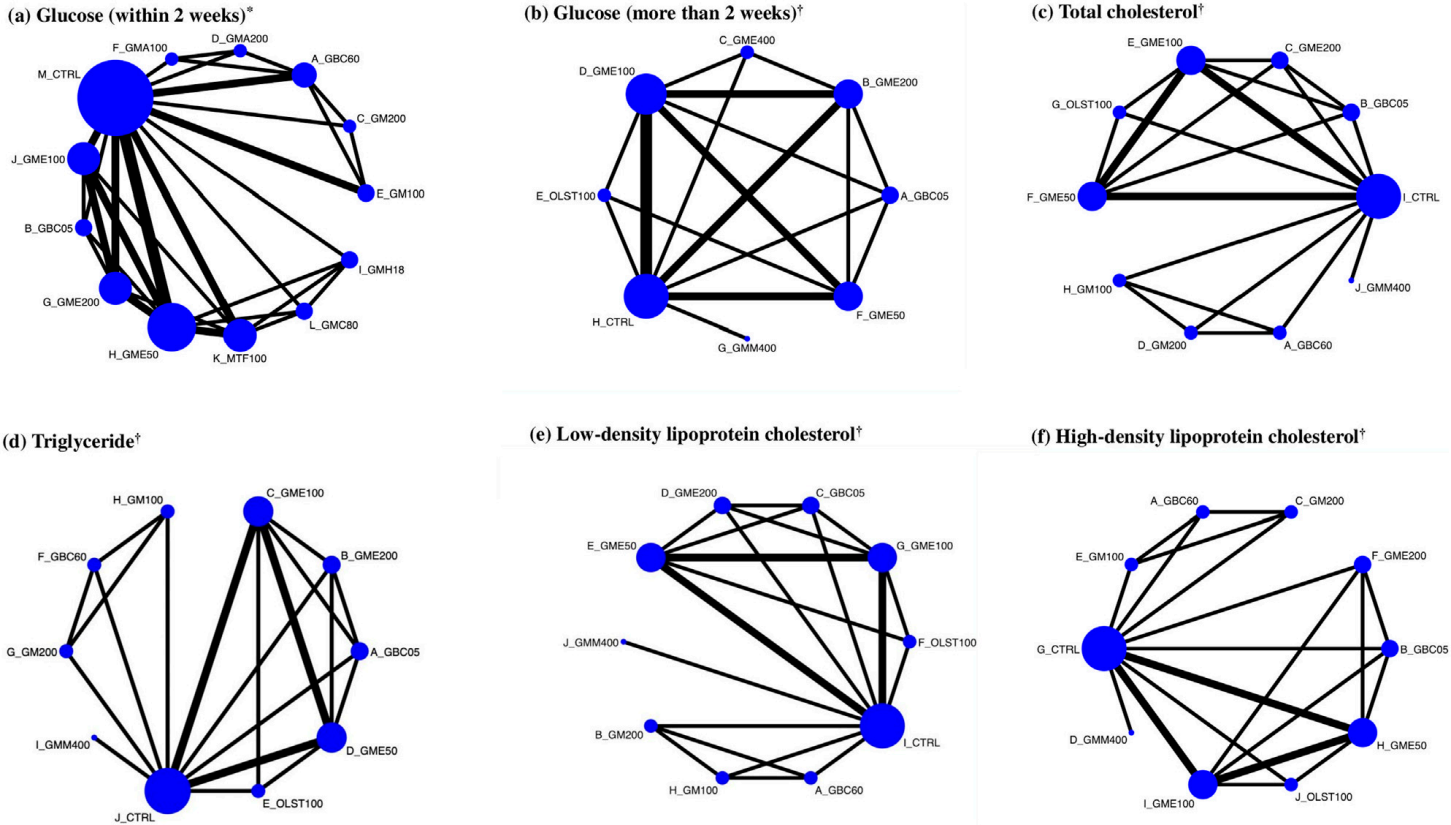
Network of eligible comparisons for glycemic control and lipid metabolism (primary and secondary outcomes) and network plots of eligible direct and indirect comparisons. Each node represents each treatment. Here, the size of the node is proportional to the number of rodents randomized to the GM treatments, and the width of the line is proportional to the number of trials comparing each pair of treatments. *Primary outcome. †Secondary outcomes. **(A)** Glucose (within 2 weeks), **(B)** glucose (more than 2 weeks), **(C)** total cholesterol, **(D)** triglyceride, **(E)** low-density lipoprotein cholesterol, and **(F)** high-density lipoprotein cholesterol.

Figure 3 illustrates the network of eligible comparisons for various outcomes, including blood glucose level within 2 weeks (six studies), blood glucose levels at more than 2 weeks (four studies), TC (four studies), TG (four studies), LDL-C (four studies), and HDL-C (four studies). The size of nodes represented the number of direct evidence for each treatment. The solid black lines connect treatment pairs within direct evidence, and the thickness of lines relates to the number of rodents within each comparison.

### 3.5 Results of the network meta-analysis

**FIGURE 4:**
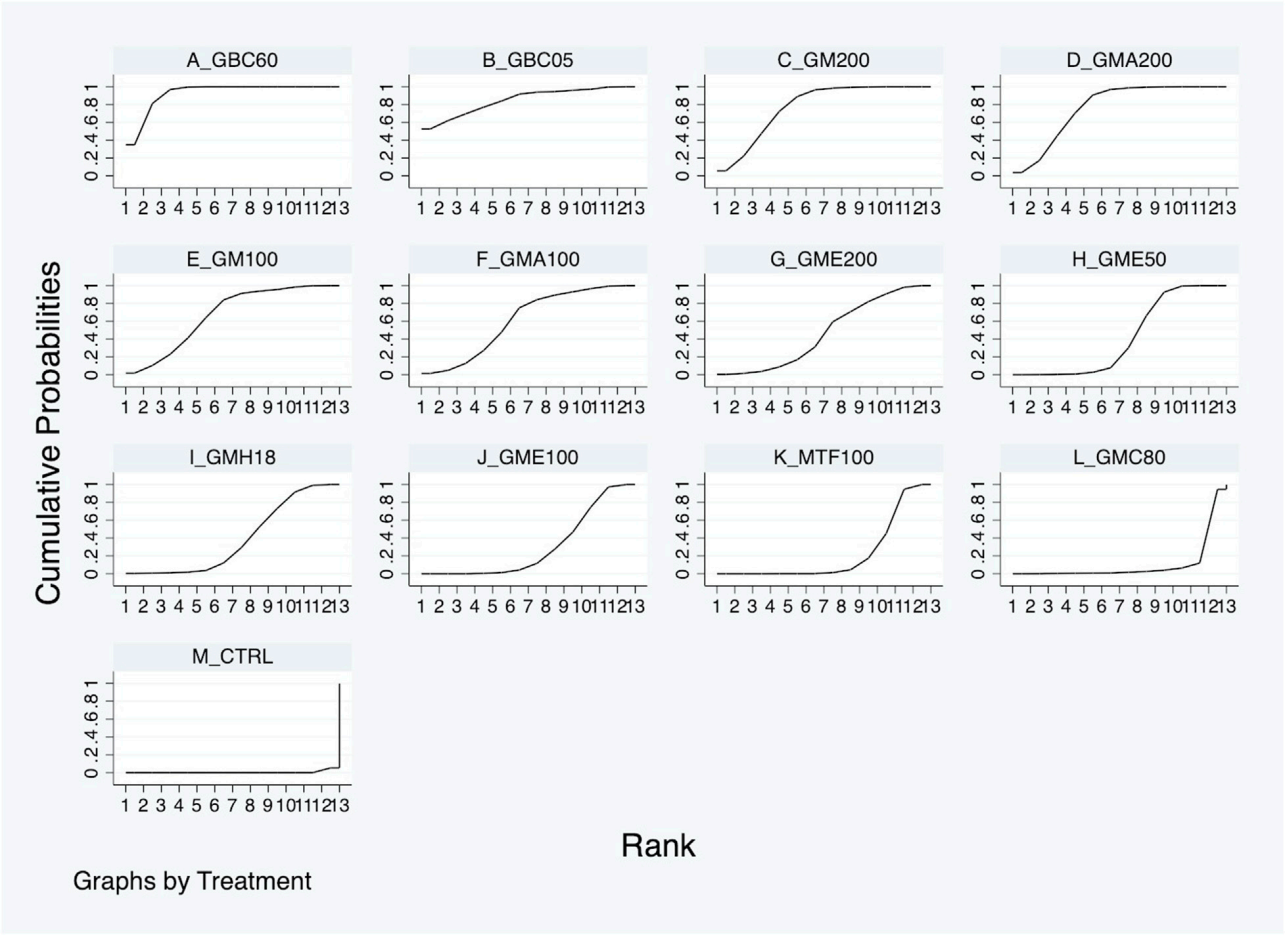
Surface under the cumulative ranking curve (SURCA) of each GM or drug treatment for blood glucose improvement within 2 weeks.

Table 2 and Supplementary Tables S5-S9 present the results of the NMA for outcomes related to glycemic control and lipid metabolism. The outcome of blood glucose levels within 2 weeks was available in six studies, as detailed in Table 2. The three most effective treatments, namely, GBC60 (WMD: −127.46 mg/dL; 95% CI: −140.22 to −114.69), GB05 (WMD: −131.49 mg/dL; 95% CI: −197.37 to −65.61), and GM200 (WMD: −116.25 mg/dL; 95% CI: −139.52 to −92.98), were more efficacious than control in decreasing blood glucose levels, as shown. Based on SUCRA, GBC60 was ranked the most effective, followed by GBC05, GM200, GMA200, GM100, GMA100, GME200, GME50, GMH18, GME100, MTF100, and GMC80 (Figure 4).

**TABLE 2:** League table showing the results of network meta-analysis comparing the effect of GM treatments in different concentrations on blood glucose level in diabetic rodent models within 2 weeks.

A_GBC60												
4.04 (−63.07, 71.14)	B_GBC05											
−11.21 (−30.87, 8.46)	−15.24 (−85.11, 54.63)	C_GM200										
−12.24 (−30.82, 6.34)	−16.27 (−84.91, 52.36)	−1.03 (−28.02, 25.96)	D_GMA200									
−19.34 (−44.52, 5.84)	−23.37 (−94.75, 48.01)	−8.13 (−37.50, 21.23)	−7.10 (38.10, 23.90)	E_GM100								
−25.56 (−52.68, 1.56)	−29.59 (−101.02, 41.83)	−14.35 (−47.80, 19.09)	−13.32 (−43.96, 17.32)	−6.22 (−42.98, 30.54)	F_GMA100							
**−41.04 (−78.30, −3.78)**	−45.08 (−111.01, 20.85)	−29.84 (−71.87, 12.19)	−28.80 (−68.75, 11.14)	−21.71 (−66.20, 22.79)	−15.48 (−60.05, 29.08)	G_GME200						
**−45.29 (−73.37, −17.21)**	−49.33 (−113.00, 14.34)	−34.09 (−68.25, 0.07)	**−33.05 (−64.61, −1.50)**	−25.96 (−63.10, 11.19)	−19.73 (−56.97, 17.50)	−4.25 (−31.03, 22.53)	H_GME50					
**−47.00 (−78.46, −15.55)**	−51.04 (−116.73, 14.65)	−35.80 (−72.78, 1.18)	**−34.76 (−69.36, −0.17)**	−27.67 (−67.43, 12.09)	−21.44 (−61.28, 18.39)	−5.96 (−37.40, 25.48)	−1.71 (−18.71, 15.28)	I_GMH18				
**−51.83 (−84.20, −19.46)**	−55.87 (−120.53, 8.80)	**−40.63 (−78.39, −2.86)**	**−39.59 (−75.02, −4.16)**	−32.49 (−72.98, 8.00)	−26.27 (−66.84, 14.29)	−10.79 (−37.83, 16.25)	−6.54 (−25.01, 11.93)	−4.83 (−29.60, 19.94)	J_GME100			
**−57.45 (−86.32, −28.58)**	−61.48 (−125.92, 2.96)	**−46.24 (−81.05, −11.43)**	**−45.21 (−77.47, −12.94)**	**−38.11 (−75.86, −0.36)**	−31.89 (−69.72, 5.94)	−16.40 (−44.98, 12.17)	−12.15 (−23.66, −0.65)	−10.44 (−28.17, 7.29)	−5.61 (−26.60, 15.37)	K_MTF100		
**−86.24 (−136.61, −35.88)**	**−90.28 (−166.85, −13.71)**	**−75.04 (−129.03, −21.04)**	**−74.00 (−126.39, −21.62)**	**−66.91 (−122.84, −10.97)**	**−60.68 (−116.67, −4.70)**	−45.20 (−95.56, 5.16)	−40.95 (−83.81, 1.91)	−39.24 (−84.12, 5.64)	−34.41 (−80.90, 12.08)	−28.80 (−71.95, 14.36)	L_GMC80	
**−127.46 (−140.22, −114.69)**	**−131.49 (−197.37, −65.61)**	**−116.25 (−139.52, −92.98)**	**−115.22 (−134.46, −95.97)**	**−108.12 (−135.59, −80.65)**	**−101.90 (−129.48, −74.32)**	**−86.41 (−121.42, −51.41)**	**−82.16 (−107.17, −57.16)**	**−80.45 (−109.20, −51.71)**	**−75.62 (−105.37, −45.88)**	**−70.01 (−95.90, −44.12)**	−41.21 (−89.94, 7.51)	M_CTRL

GM and drug treatments are ranked in order.

Number below each treatment represents the mean difference (95% confidence interval) in the blood glucose level (mg/dL) between the row and the column.

Note: GBC05, glibenclamide at 0.5 mg/kg body weight; GBC60, glibenclamide at 60 mg/kg body weight; GM100, Garcinia mangostana extract at 100 mg/kg body weight; GM200, Garcinia mangostana extract at 200 mg/kg body weight; GMA100, aqueous extract of Garcinia mangostana at 100 mg/kg body weight; GMA200, aqueous extract of Garcinia mangostana at 200 mg/kg body weight; GMC80, chloroform extract of Garcinia mangostana at 80 mg/kg body weight; GME50, ethanol extract of Garcinia mangostana at 50 mg/kg body weight; GME100, ethanol extract of *Garcinia mangostana* at 100 mg/kg body weight; GME200, ethanol extract of *Garcinia mangostana* at 200 mg/kg body weight; GMH18, hexane extract of *Garcinia mangostana* at 18 mg/kg body weight; MTF100, metformin at 100 mg/kg body weight; CTRL, diabetic control. Bold values indicate statistical significance.

In comparison to the diabetic control group for a duration more than 2 weeks, GBC05 revealed significant effectiveness in reducing the blood glucose level (WMD: −316.50 mg/dL; 95% CI: −519.59 to −113.42), followed by GME200 (WMD: −216.37 mg/dL; 95% CI: −371.27 to −61.47), GME400 (WMD: −217.07 mg/dL; 95% CI: −421.98 to −12.15), and GME100 (MD: −186.43 mg/dL; 95% CI: −319.75 to −53.11), as shown in Supplementary Table S5. Although OLST100, GME50, and GMM400 showed some tendency toward better performance than the diabetic control group, their effects were not statistically significant. All treatments (GBC60, GME200, GME400, GME100, OLST100, GME50, and GMM400) were ranked based on their SURCA, as illustrated in Supplementary Figure S1.

For the lipid metabolism improvement, the three most effective treatments, including GBC60 (WMD: −108.00 mg/dL; 95% CI: −128.12 to −87.88), GBC05 (WMD: −63.15 mg/dL; 95% CI: −71.89 to −55.13), and GME200 (WMD: −60.84 mg/dL; 95% CI: −70.09 to −51.59), demonstrated greater effectiveness than the diabetic control group in reducing the total cholesterol level (see Supplementary Table S6). According to the SURCA, GBC60 was ranked the most effective in reducing TC, followed by GBC05, GME200, GM200, GME100, GME50, OLST100, and GM100 (see Supplementary Figure S2). For triglyceride reduction, GBC50 (WMD: −116.22 mg/dL; 95% CI: −154.00 to −78.45), GME200 (WMD: −87.22 mg/dL; 95% CI: −124.98 to −49.47), and GME100 (WMD: −79.38 mg/dL; 95% CI: −110.59 to −48.18) were identified as the three most effective treatments, as indicated in Supplementary Table S7. GBC05 was ranked as the most effective in decreasing triglyceride levels, followed by GME200, GME100, GME50, OLST1000, GBC50, GM200, GM100, and GMM400, according to Supplementary Figure S3.

In comparison to the diabetic control group, the outcomes of LDL-C were available in four studies (see Figure 3). GBC60 (WMD: −110.00 mg/dL; 95% CI: −136.37 to −83.63), GMM200 (WMD: −58.00 mg/dL; 95% CI: −82.63 to −33.37), GBC05 (WMD: −54.07 mg/dL; 95% CI: −72.64 to −35.49), and GME200 (WMD: −52.45 mg/dL; 95% CI: −71.31 to −33.58) were identified as the three most effective treatments, whereas GM100 and GMM400 had no significant treatment effect compared with diabetic control (Supplementary Table S8). Based on the SURCA, GBC50 was ranked as the most effective, followed by GM200, GBC05, GME200, GME50, OLST100, GME100, GM100, and GMM400, as shown in Supplementary Figure S4. The three most effective treatments for increasing HDL-C levels were GBC60 (WMD: 11.00 mg/dL; 95% CI: −1.74–23.74), GBC05 (WMD: 5.26 mg/dL; 95% CI: −6.80–17.32), and GM200 (WMD: 5.00 mg/dL; 95% CI: −7.66–17.66 mg/dL) (see Supplementary Table S9). GBC60 was ranked the most effective for HDL elevation based on the SURCA, followed by GBC05, GM200, GMM400, GM100, and GME200, whereas GME50, GME100, and OLST100 were found to be less effective than the diabetic control (see Supplementary Figure S5).

### 3.6 Sensitivity analysis

Sensitivity analysis, by removing the studied with imputed SD, showed slighted changes in treatment, indicating the robustness of the primary analysis, as shown in Supplementary Table S10.

### 3.7 Strength of evidence assessment

All pairwise treatment comparisons of the primary outcome were assessed for the strength of evidence, and the results are presented in Supplementary Table S7. Most domains, including within-study bias, reporting bias, indirectness, and incoherence, were rated as “No concerns.” However, the proportion of pairwise comparisons rated as “Some concerns” and “Major concerns” was the highest in imprecision, followed by heterogeneity (see Supplementary Table S11). The heterogeneity in this network meta-analysis appears to be manageable, as most comparisons indicate a low risk of bias with “No concerns.” However, areas with “Some concerns” should be addressed with caution. When there are very few trials, as in this study, the estimation of heterogeneity is limited, and the confidence intervals may be unreliable (Turner et al., 2012). Overall, the confidence rating for the estimated treatment effect of primary outcome was graded as moderate-to-high confidence.

## 4 Discussion

DM is a metabolic disease characterized by hyperglycemia, which results from insufficient insulin secretion, resistance to insulin action, or a combination of both factors. The abnormalities in carbohydrate, fat, and protein metabolism in diabetes are attributed to the deficient action of insulin on target tissues (Mills et al., 2022). The current oral treatment options for T2DM are categorized into ten classes: 1) sulfonylureas, 2) meglitinides, 3) metformin (a biguanide), 4) thiazolidinediones (TZDs), 5) alpha-glucosidase inhibitors, 6) dipeptidyl peptidase IV (DPP-4) inhibitors, 7) bile acid sequestrants, 8) dopamine agonists, 9) sodium-glucose transport protein 2 (SGLT2) inhibitors, and 10) oral glucagon-like peptide 1 (GLP-1) receptor agonists. In addition, GLP-1 receptor agonists, dual GLP-1 and glucose-dependent insulinotropic peptide (GIP) receptor agonists, and amylin can be administered by injection (Babiker and Al Dubayee, 2017). Metformin, an oral hypoglycemic agent, remains the first-line treatment for type 2 DM but is associated with adverse effects such as gastrointestinal disturbances (such as nausea, vomiting, and diarrhea) and lactic acidosis (Blough et al., 2015; McCreight et al., 2016). Given these limitations, there is a growing interest in natural products as alternative therapies due to their perceived safety, efficacy, and cost-effectiveness.

*Garcinia mangostana* L., a member of the Clusiaceae family, is typically found in Southeast Asian countries such as Thailand, Malaysia, and Indonesia (Bi et al., 2023). In addition to its effects on blood sugar levels, mangosteen peels provide various general health benefits due to their rich content of bioactive compounds. Mangosteen juice has been shown to reduce inflammation and promote weight loss (Udani et al., 2009). Moreover, mangosteen extract significantly improved insulin resistance, weight management, and inflammatory status in obese female patients with insulin resistance (Watanabe et al., 2018). A 30-day consumption of a mangosteen-based drink was also found to enhance antioxidant and anti-inflammatory biomarkers in healthy adults (Xie et al., 2015). Mangosteen peels have a wide range of biologically active metabolites such as xanthones, isoflavones, tannins, flavonoids, procyanidin, benzophenones, and α-, β-, and γ-mangostin (Songsiriritthigul et al., 2020; Tran et al., 2021; Zheng et al., 2021). Notably, xanthones emerge as the major phytochemical metabolites. α-Mangostin, isolated from mangosteen peel and pericarp, exhibits a broad spectrum of biological and pharmacological activities, including anti-inflammatory, antioxidant, anti-cancer, anti-obesity, anti-diabetic, anti-bacterial, anti-ulcerogenic, and anti-angiogenic activities, in addition to cardioprotective, hepatoprotective, and neuroprotective effects (Devi Sampath and Vijayaraghavan, 2007; Gutierrez-Orozco et al., 2013; Shiozaki et al., 2013; Hafeez et al., 2014; Janhom and Dharmasaroja, 2015; Tsai et al., 2016; Fu et al., 2018; Sivaranjani et al., 2019; Usman et al., 2021). Another significant metabolite, γ-mangostin, is a xanthone derivative isolated from the fruit hull of mangosteen which has anti-cancer, anti-hyperglycemic, and anti-leptospiral activities (Chang and Yang, 2012; Seesom et al., 2013; Chen et al., 2021). Although previous studies and reviews have suggested the anti-diabetic potential of *G. mangostana* extracts, a comprehensive systematic review and network meta-analysis (NMA) evaluating its effect on DM has not been conducted (Tousian Shandiz et al., 2017; Rizaldy et al., 2022). This study aimed to fill this gap by performing an NMA to evaluate and compare the hypoglycemic activity of GM extracts.

The present systematic review and first NMA aimed to evaluate the hypoglycemic activity of *G. mangostana* L. (GM) extracts on DM. A comprehensive literature search resulted in 306 articles, from which 10 studies were selected in the final quantitative synthesis (as shown in Figure 1). These studies, published since 2016, utilized various parts of the GM plant, different solvents for extraction, and diverse rodent models to investigate the effects of GM extracts on DM (summarized in Table 1). The NMA results revealed significant improvements in glycemic control with GM extracts compared to controls. The network plot in Figure 3 illustrates the comparisons made among different treatments. For blood glucose levels within 2 weeks, the most effective treatments were GBC60, GBC05, and GM200, which showed substantial reductions in blood glucose levels (as depicted in Table 2. The SUCRA values indicated that GBC60 had the highest effectiveness, followed by GBC05 and GM200, as summarized in Figure 4. For blood glucose levels more than 2 weeks, GBC05, GME200, GME400, and GME100 were the most effective treatments, as summarized in Supplementary Figure S1. The results suggest that GM extracts have a sustained hypoglycemic effect over longer durations.

The analysis of lipid profiles revealed that GM extracts also positively affected the lipid metabolism. The most effective treatments for reducing total cholesterol levels were GBC60, GBC05, and GME200, as summarized in Supplementary Table S6. Similarly, for triglyceride levels, GBC50, GME200, and GME100 showed the greatest effectiveness (depicted in Supplementary Table S7). The reduction in LDL-C levels was most significant with GBC60, GMM200, GBC05, and GME200 (shown in Supplementary Table S8). Additionally, the treatments with GBC60, GBC05, and GM200 were identified as the most effective for increasing HDL-C levels (shown in Supplementary Table S9). The risk of bias assessment, as shown in Figure 2, indicated that the included studies generally had a low risk of bias. The strength of evidence, evaluated using the CINeMA approach, was rated as moderate to high for most outcomes, with some concerns noted in domains such as imprecision and heterogeneity, as summarized in Supplementary Table S11.

GME from mangosteen peel demonstrated a significant decrease in glucose, TG, TC, LDL-C, and free fatty acid levels in mice subjected to a high-fat diet. Additionally, GME treatment activated the hepatic AMP-activated protein kinase and Sirtuin 1, which showed anti-obesity effects (Chae et al., 2016). Similarly, GME from mangosteen pericarp exhibited notable effects, including decreased fasting blood glucose and malondialdehyde levels, increased body weight, improving the islets of Langerhans, and activated insulin secretion in STZ-induced diabetic mice (Husen et al., 2019). Furthermore, GME from pericarp was able to reduce serum interleukin-6 (IL-6) and tumor necrosis factor-α (TNF-α) levels, increase superoxide dismutase (SOD) activity, and reduce hippocampal nuclear factor-κB (NF-κB), IL-6, and TNF-α in obese type-2 diabetes mellitus Wistar rats (Muniroh et al., 2021). GME from pericarp also significantly decreased the levels of TG, TC, low-density lipoprotein (LDL), very low-density lipoprotein (VLDL), serum glutamic oxaloacetic transaminase (SGOT), serum glutamic pyruvic transaminase (SGPT), urea, and creatinine compared to STZ-diabetic rats (Taher et al., 2016). GMH, GMC, and GME from pericarps significantly reduced the levels of fasting blood glucose and HbA1c in diabetic mice (Husen et al., 2017). Treatment with GM from mangosteen vinegar rind (MVR) in HFD/STZ-induced diabetic mice resulted in a significant decrease in plasma glucose, plasma lipid profile (TC, TG, and LDL), hepatic lipid profile (TC and TG in liver tissue), and liver function tests (aspartate aminotransferase (AST) and alanine aminotransferase (ALT)), along with reduced oxidative stress markers (bilirubin and MDA). Moreover, GM from MVR markedly increased plasma HDL, SOD, and catalase (CAT) levels (Karim et al., 2017). GMA from mangosteen pericarp treatment in HFD and STZ-induced diabetic mice significantly reduced fasting plasma glucose level, α-amylase activity, renal function parameters (kidney hypertrophy, blood urea nitrogen, and creatinine), and antioxidant parameters (MDA), whereas GME improved SOD and CAT levels, restored kidney architectural damage, and decreased the apoptotic cells of kidney in diabetic mice (Karim et al., 2019). GMM from mangosteen pericarp significantly decreased blood glucose levels and body mass index (BMI) in obese rats (Labban et al., 2021). GM from mangosteen pericarp lowered the blood glucose level and increased spermatogenesis in STZ-induced diabetic rats (Primiani and Lestari, 2019). Findings from various studies indicate mangosteen extracts possess anti-diabetic potential by improving blood glucose levels, lipid profiles, oxidative stress, and hepatic functions.

The benefit of conducting an NMA in this study is that it allows for a comprehensive evaluation and comparison of the hypoglycemic effects of various GM extracts on diabetes mellitus. By synthesizing data from multiple studies, the NMA provides a clearer understanding of the relative effectiveness of different GM extract treatments on blood glucose levels and lipid profiles. This approach allows for the identification of the most effective treatments, such as GM200, as well as the sustained hypoglycemic effects over longer durations. Additionally, the NMA addresses variations in study designs, such as the use of different parts of the GM plant, solvents, and rodent models, ensuring that the conclusions drawn are robust and applicable across diverse scenarios. Overall, the NMA offers a powerful tool for guiding the selection of GM extracts in managing DM by providing a systematic and quantitative assessment of their effectiveness.

The findings of this study hold significant implications for the development of natural product-based therapies for diabetes. The hypoglycemic effects demonstrated by GM extracts suggest the potential for integrating such natural products into diabetes treatment protocols, particularly as alternatives or supplements to conventional therapies like metformin. Given the challenges associated with current pharmacological treatments, such as side effects and cost, GM extracts offer a promising, safer, and potentially more cost-effective solution. Additionally, the antioxidant, anti-inflammatory, and lipid-lowering properties of GM extracts could provide broader therapeutic benefits beyond glycemic control, addressing other metabolic complications common in diabetes patients, such as dyslipidemia and oxidative stress. However, applying these findings into human treatments requires further research, including clinical trials, to determine safety, efficacy, optimal dosing, and potential long-term effects. These broader implications highlight the role of natural products in advancing diabetes treatment, especially in regions where traditional medicine plays a significant role in health-care practices.

### 4.1 Strength and limitation of the study

Animal models serve as a basis for the design of pharmacology research and later studies in humans. Ideally, these models should be applied to represent the diversity in human diabetic patients (King, 2012). Our study is the first NMA investigating the effects of GM and GM extracts on diabetes in rodent models. Systematic review and meta-analysis amalgamate findings from animal experiments, shedding light on the effectiveness or side effects of a treatment or intervention, thereby offering valuable insights for further human studies (Bahadoran et al., 2020). Although clinical data on the pharmacological effects of GM and GM extracts in diabetes treatment are limited, their therapeutic potential is supported by the hypoglycemic effects observed in this NMA. Furthermore, the anti-diabetic effects may not be limited to blood glucose levels but also extend to improvements in TC, TG, LDL-C, and HDL-C. Traditional medicines derived from plant extracts have demonstrated effectiveness in preventing and treating DM, attributed to the presence of numerous bioactive phytochemical metabolites with diverse beneficial biological effects and minimal side effects (Usai et al., 2022).

However, there were significant variations in the study designs, dosages, and animal models used across the included studies. The studies utilized different parts of the *G. mangostana* L. plant, employed various extraction methods, and involved diverse rodent models, including different species and diabetes induction methods. The dosages of GM extracts also varied, ranging from 18 to 400 mg/kg body weight. These differences could potentially influence the outcomes and add heterogeneity to the meta-analysis. Additionally, some studies had small sample sizes, which could affect the reliability of the findings. Differences in study quality, such as varying risk of bias levels, could also contribute to inconsistencies in the results. Addressing these issues provides a more nuanced interpretation of the findings and highlights the need for further research with more standardized designs and larger sample sizes to validate the results. Therefore, additional experimental and evidence-based studies are necessary to determine the effects of plant extracts in managing DM, as well as to explore the molecular mechanisms of GM and its extracts.

## 5 Conclusion

Overall, GM and its extracts exhibited promising anti-diabetic effects and demonstrated an ability to improve lipid profiles in rodent diabetic models. These findings support the potential of GM extracts as a complementary treatment for DM. However, the studies included in this analysis were limited to animal models, and further research is needed to explore the anti-diabetic effects of GM and its extracts in human subjects. Although this study offers valuable references for subsequent research and clinical trials, further clinical studies are strongly recommended to validate the anti-diabetic effects of GM and its extracts. This validation process will contribute to the development of suitable formulations for diabetes treatment.

## Data Availability

The raw data supporting the conclusions of this article will be made available by the authors, without undue reservation.
