# Supplementary material for: Hypoglycemic activity of Garcinia mangostana L. extracts on diabetes rodent models: A systematic review and network meta-analysis
Source: Front Pharmacol. 2024 Oct 2;15:1472419. doi: 10.3389/fphar.2024.1472419 (PMC11479905; doi:10.3389/fphar.2024.1472419)
Supplement: Supplementary file 1 [file DataSheet1.docx]

**Supplementary Materials**

**catalogue**

| **Table S1.** PRISMA NMA checklist of items to include when reporting a systematic review involving a network meta-analysis. | 2 |
| --- | --- |
| **Table S2.** Database search strategies from PubMed database. | 6 |
| **Table S3.** Database search strategies from Embase database. | 12 |
| **Table S4.** Database search strategies from Scopus database. | 13 |
| **Table S5.**  League table showing the results of network meta-analysis comparing the effect of GM treatments in different concentrations or drug treatment on blood glucose level in diabetic rodent models at more than 2 weeks. | 14 |
| **Table S6.**  League table showing the results of network meta-analysis comparing the effect of GM treatments in different concentrations or drug treatment on total cholesterol in diabetic rodent models. | 15 |
| **Table S7.**  League table showing the results of network meta-analysis comparing the effect of GM treatments in different concentrations or drug treatment on triglyceride levels in diabetic rodent models. | 16 |
| **Table S8.** League table showing the results of network meta-analysis comparing the effect of GM treatments in different concentrations or drug treatment on LDL-C levels in diabetic rodent models. | 17 |
| **Table S9.**  League table showing the results of network meta-analysis comparing the effect of GM treatments in different concentrations or drug treatment on HDL-C levels in diabetic rodent models. | 18 |
| **Table S10**. Sensitivity analyses according to the blood glucose measured at more than 2 weeks | 19 |
| **Table S11.** CINeMa framework confidence ratings for each available pairwise comparison. | 20 |
| **Figure S1.** The surface under the cumulative ranking curve (SURCA) of each GM or drug treatment for blood glucose improvement at more than 2 weeks. | 29 |
| **Figure S2.** The surface under the cumulative ranking curve (SURCA) of each GM or drug treatment for total cholesterol improvement. | 30 |
| **Figure S3**. The surface under the cumulative ranking curve (SURCA) of each GM or drug treatment for triglyceride improvement. | 31 |
| **Figure S4.** The surface under the cumulative ranking curve (SURCA) of each GM or drug treatment for LDL-C improvement. | 32 |
| **Figure S5.** The surface under the cumulative ranking curve (SURCA) of each GM or drug treatment for HDL-C improvement. | 33 |

**Table S1.** PRISMA NMA checklist of items to include when reporting a systematic review involving a network meta-analysis.

| **Section/Topic** | **Item #** | **Checklist Item** | **Checklist** |
| --- | --- | --- | --- |
| **TITLE** |  |  |  |
| Title | 1 | Identify the report as a systematic review *incorporating a network meta-analysis (or related form of meta-analysis).* | ☑ |
|  |  |  |  |
| **ABSTRACT** |  |  |  |
| Structured summary | 2 | Provide a structured summary including, as applicable:  **Background:** main objectives  **Methods:** data sources; study eligibility criteria, participants, and interventions; study appraisal; and *synthesis methods, such as network meta-analysis.*  **Results:** number of studies and participants identified; summary estimates with corresponding confidence/credible intervals; *treatment rankings may also be discussed. Authors may choose to summarize pairwise comparisons against a chosen treatment included in their analyses for brevity.*  **Discussion/Conclusions:** limitations; conclusions and implications of findings.  **Other:** primary source of funding; systematic review registration number with registry name. | ☑ |
|  |  |  |  |
| **INTRODUCTION** |  |  |  |
| Rationale | 3 | Describe the rationale for the review in the context of what is already known*, including mention of why a network meta-analysis has been conducted.* | ☑ |
| Objectives | 4 | Provide an explicit statement of questions being addressed, with reference to participants, interventions, comparisons, outcomes, and study design (PICOS). | ☑ |
|  |  |  |  |
| **METHODS** |  |  |  |
| Protocol and registration | 5 | Indicate whether a review protocol exists and if and where it can be accessed (e.g., Web address); and, if available, provide registration information, including registration number. | ☑ |
| Eligibility criteria | 6 | Specify study characteristics (e.g., PICOS, length of follow-up) and report characteristics (e.g., years considered, language, publication status) used as criteria for eligibility, giving rationale. *Clearly describe eligible treatments included in the treatment network, and note whether any have been clustered or merged into the same node (with justification).* | ☑ |
| Information sources | 7 | Describe all information sources (e.g., databases with dates of coverage, contact with study authors to identify additional studies) in the search and date last searched. | ☑ |
| Search | 8 | Present full electronic search strategy for at least one database, including any limits used, such that it could be repeated. | ☑ |
| Study selection | 9 | State the process for selecting studies (i.e., screening, eligibility, included in systematic review, and, if applicable, included in the meta-analysis). | ☑ |
| Data collection process | 10 | Describe method of data extraction from reports (e.g., piloted forms, independently, in duplicate) and any processes for obtaining and confirming data from investigators. | ☑ |
| Data items | 11 | List and define all variables for which data were sought (e.g., PICOS, funding sources) and any assumptions and simplifications made. | ☑ |
| **Geometry of the network** | **S1** | Describe methods used to explore the geometry of the treatment network under study and potential biases related to it. This should include how the evidence base has been graphically summarized for presentation, and what characteristics were compiled and used to describe the evidence base to readers. | ☑ |
| Risk of bias within individual studies | 12 | Describe methods used for assessing risk of bias of individual studies (including specification of whether this was done at the study or outcome level), and how this information is to be used in any data synthesis. | ☑ |
| Summary measures | 13 | State the principal summary measures (e.g., risk ratio, difference in means). *Also describe the use of additional summary measures assessed, such as treatment rankings and surface under the cumulative ranking curve (SUCRA) values, as well as modified approaches used to present summary findings from meta-analyses.* | ☑ |
| Planned methods of analysis | 14 | Describe the methods of handling data and combining results of studies for each network meta-analysis. This should include, but not be limited to:   - *Handling of multi-arm trials;* - *Selection of variance structure;* - *Selection of prior distributions in Bayesian analyses; and* - *Assessment of model fit.* | ☑ |
| **Assessment of Inconsistency** | **S2** | Describe the statistical methods used to evaluate the agreement of direct and indirect evidence in the treatment network(s) studied. Describe efforts taken to address its presence when found. | ☑ |
| Risk of bias across studies | 15 | Specify any assessment of risk of bias that may affect the cumulative evidence (e.g., publication bias, selective reporting within studies). | ☑ |
| Additional analyses | 16 | Describe methods of additional analyses if done, indicating which were pre-specified. This may include, but not be limited to, the following:   - Sensitivity or subgroup analyses; - Meta-regression analyses; - *Alternative formulations of the treatment network; and* - *Use of alternative prior distributions for Bayesian analyses (if applicable).* | ☑ |
|  |  |  |  |
| **RESULTS†** |  |  |  |
| Study selection | 17 | Give numbers of studies screened, assessed for eligibility, and included in the review, with reasons for exclusions at each stage, ideally with a flow diagram. | ☑ |
| **Presentation of network structure** | **S3** | Provide a network graph of the included studies to enable visualization of the geometry of the treatment network. | ☑ |
| **Summary of network geometry** | **S4** | Provide a brief overview of characteristics of the treatment network. This may include commentary on the abundance of trials and randomized patients for the different interventions and pairwise comparisons in the network, gaps of evidence in the treatment network, and potential biases reflected by the network structure. | ☑ |
| Study characteristics | 18 | For each study, present characteristics for which data were extracted (e.g., study size, PICOS, follow-up period) and provide the citations. | ☑ |
| Risk of bias within studies | 19 | Present data on risk of bias of each study and, if available, any outcome level assessment. | ☑ |
| Results of individual studies | 20 | For all outcomes considered (benefits or harms), present, for each study: 1) simple summary data for each intervention group, and 2) effect estimates and confidence intervals. *Modified approaches may be needed to deal with information from larger networks.* |  |
| Synthesis of results | 21 | Present results of each meta-analysis done, including confidence/credible intervals. *In larger networks, authors may focus on comparisons versus a particular comparator (e.g. placebo or standard care), with full findings presented in an appendix. League tables and forest plots may be considered to summarize pairwise comparisons.* If additional summary measures were explored (such as treatment rankings), these should also be presented. | ☑ |
| **Exploration for inconsistency** | **S5** | Describe results from investigations of inconsistency. This may include such information as measures of model fit to compare consistency and inconsistency models, *P* values from statistical tests, or summary of inconsistency estimates from different parts of the treatment network. | ☑ |
| Risk of bias across studies | 22 | Present results of any assessment of risk of bias across studies for the evidence base being studied. | ☑ |
| Results of additional analyses | 23 | Give results of additional analyses, if done (e.g., sensitivity or subgroup analyses, meta-regression analyses*, alternative network geometries studied, alternative choice of prior distributions for Bayesian analyses,* and so forth). | ☑ |
|  |  |  |  |
| **DISCUSSION** |  |  |  |
| Summary of evidence | 24 | Summarize the main findings, including the strength of evidence for each main outcome; consider their relevance to key groups (e.g., healthcare providers, users, and policy-makers). | ☑ |
| Limitations | 25 | Discuss limitations at study and outcome level (e.g., risk of bias), and at review level (e.g., incomplete retrieval of identified research, reporting bias). *Comment on the validity of the assumptions, such as transitivity and consistency. Comment on any concerns regarding network geometry (e.g., avoidance of certain comparisons).* | ☑ |
| Conclusions | 26 | Provide a general interpretation of the results in the context of other evidence, and implications for future research. | ☑ |
|  |  |  |  |
| **FUNDING** |  |  |  |
| Funding | 27 | Describe sources of funding for the systematic review and other support (e.g., supply of data); role of funders for the systematic review. This should also include information regarding whether funding has been received from manufacturers of treatments in the network and/or whether some of the authors are content experts with professional conflicts of interest that could affect use of treatments in the network. | ☑ |

PICOS = population, intervention, comparators, outcomes, study design.

* Text in italics indicateS wording specific to reporting of network meta-analyses that has been added to guidance from the PRISMA statement.

† Authors may wish to plan for use of appendices to present all relevant information in full detail for items in this section.

**Table S2.** Database search strategies from PubMed database.

| **Search number** | **Query** | **Search Details** | **Results** |
| --- | --- | --- | --- |
| 1 | garcinia mangostana | "garcinia mangostana"[MeSH Terms] OR ("garcinia"[All Fields] AND "mangostana"[All Fields]) OR "garcinia mangostana"[All Fields] | 604 |
| 2 | G. mangostana | "g"[All Fields] AND "mangostana"[All Fields] | 157 |
| 3 | mangosteen | "garcinia mangostana"[MeSH Terms] OR ("garcinia"[All Fields] AND "mangostana"[All Fields]) OR "garcinia mangostana"[All Fields] OR "mangosteen"[All Fields] | 767 |
| 4 | Garcinia mangostana extract | ("garcinia mangostana"[MeSH Terms] OR ("garcinia"[All Fields] AND "mangostana"[All Fields]) OR "garcinia mangostana"[All Fields]) AND ("extract"[All Fields] OR "extract s"[All Fields] OR "extractabilities"[All Fields] OR "extractability"[All Fields] OR "extractable"[All Fields] OR "extractables"[All Fields] OR "extractant"[All Fields] OR "extractants"[All Fields] OR "extracted"[All Fields] OR "extractibility"[All Fields] OR "extractible"[All Fields] OR "extracting"[All Fields] OR "extraction"[All Fields] OR "extractions"[All Fields] OR "extractive"[All Fields] OR "extractives"[All Fields] OR "extracts"[All Fields]) | 342 |
| 5 | G. mangostana extract | "g"[All Fields] AND "mangostana"[All Fields] AND ("extract"[All Fields] OR "extract s"[All Fields] OR "extractabilities"[All Fields] OR "extractability"[All Fields] OR "extractable"[All Fields] OR "extractables"[All Fields] OR "extractant"[All Fields] OR "extractants"[All Fields] OR "extracted"[All Fields] OR "extractibility"[All Fields] OR "extractible"[All Fields] OR "extracting"[All Fields] OR "extraction"[All Fields] OR "extractions"[All Fields] OR "extractive"[All Fields] OR "extractives"[All Fields] OR "extracts"[All Fields]) | 105 |
| 6 | mangosteen extract | ("garcinia mangostana"[MeSH Terms] OR ("garcinia"[All Fields] AND "mangostana"[All Fields]) OR "garcinia mangostana"[All Fields] OR "mangosteen"[All Fields]) AND ("extract"[All Fields] OR "extract s"[All Fields] OR "extractabilities"[All Fields] OR "extractability"[All Fields] OR "extractable"[All Fields] OR "extractables"[All Fields] OR "extractant"[All Fields] OR "extractants"[All Fields] OR "extracted"[All Fields] OR "extractibility"[All Fields] OR "extractible"[All Fields] OR "extracting"[All Fields] OR "extraction"[All Fields] OR "extractions"[All Fields] OR "extractive"[All Fields] OR "extractives"[All Fields] OR "extracts"[All Fields]) | 407 |
| 7 | diabetes mellitus | "diabetes mellitus"[MeSH Terms] OR ("diabetes"[All Fields] AND "mellitus"[All Fields]) OR "diabetes mellitus"[All Fields] | 594,988 |
| 8 | glucose | "glucose"[MeSH Terms] OR "glucose"[All Fields] OR "glucoses"[All Fields] OR "glucose s"[All Fields] | 665,507 |
| 9 | hyperglycemia | "hyperglycaemia"[All Fields] OR "hyperglycemia"[MeSH Terms] OR "hyperglycemia"[All Fields] OR "hyperglycaemias"[All Fields] OR "hyperglycemias"[All Fields] OR "hyperglycemia s"[All Fields] | 84,675 |
| 10 | #1 OR #2 OR #3 OR #4 OR #5 OR #6 | "garcinia mangostana"[MeSH Terms] OR ("garcinia"[All Fields] AND "mangostana"[All Fields]) OR "garcinia mangostana"[All Fields] OR ("g"[All Fields] AND "mangostana"[All Fields]) OR ("garcinia mangostana"[MeSH Terms] OR ("garcinia"[All Fields] AND "mangostana"[All Fields]) OR "garcinia mangostana"[All Fields] OR "mangosteen"[All Fields]) OR (("garcinia mangostana"[MeSH Terms] OR ("garcinia"[All Fields] AND "mangostana"[All Fields]) OR "garcinia mangostana"[All Fields]) AND ("extract"[All Fields] OR "extract s"[All Fields] OR "extractabilities"[All Fields] OR "extractability"[All Fields] OR "extractable"[All Fields] OR "extractables"[All Fields] OR "extractant"[All Fields] OR "extractants"[All Fields] OR "extracted"[All Fields] OR "extractibility"[All Fields] OR "extractible"[All Fields] OR "extracting"[All Fields] OR "extraction"[All Fields] OR "extractions"[All Fields] OR "extractive"[All Fields] OR "extractives"[All Fields] OR "extracts"[All Fields])) OR ("g"[All Fields] AND "mangostana"[All Fields] AND ("extract"[All Fields] OR "extract s"[All Fields] OR "extractabilities"[All Fields] OR "extractability"[All Fields] OR "extractable"[All Fields] OR "extractables"[All Fields] OR "extractant"[All Fields] OR "extractants"[All Fields] OR "extracted"[All Fields] OR "extractibility"[All Fields] OR "extractible"[All Fields] OR "extracting"[All Fields] OR "extraction"[All Fields] OR "extractions"[All Fields] OR "extractive"[All Fields] OR "extractives"[All Fields] OR "extracts"[All Fields])) OR (("garcinia mangostana"[MeSH Terms] OR ("garcinia"[All Fields] AND "mangostana"[All Fields]) OR "garcinia mangostana"[All Fields] OR "mangosteen"[All Fields]) AND ("extract"[All Fields] OR "extract s"[All Fields] OR "extractabilities"[All Fields] OR "extractability"[All Fields] OR "extractable"[All Fields] OR "extractables"[All Fields] OR "extractant"[All Fields] OR "extractants"[All Fields] OR "extracted"[All Fields] OR "extractibility"[All Fields] OR "extractible"[All Fields] OR "extracting"[All Fields] OR "extraction"[All Fields] OR "extractions"[All Fields] OR "extractive"[All Fields] OR "extractives"[All Fields] OR "extracts"[All Fields])) | 769 |
| 11 | #7 OR #8 OR #9 | "diabetes mellitus"[MeSH Terms] OR ("diabetes"[All Fields] AND "mellitus"[All Fields]) OR "diabetes mellitus"[All Fields] OR ("glucose"[MeSH Terms] OR "glucose"[All Fields] OR "glucoses"[All Fields] OR "glucose s"[All Fields]) OR ("hyperglycaemia"[All Fields] OR "hyperglycemia"[MeSH Terms] OR "hyperglycemia"[All Fields] OR "hyperglycaemias"[All Fields] OR "hyperglycemias"[All Fields] OR "hyperglycemia s"[All Fields]) | 1,091,851 |
| 12 | #10 and #11 | ("garcinia mangostana"[MeSH Terms] OR ("garcinia"[All Fields] AND "mangostana"[All Fields]) OR "garcinia mangostana"[All Fields] OR ("g"[All Fields] AND "mangostana"[All Fields]) OR ("garcinia mangostana"[MeSH Terms] OR ("garcinia"[All Fields] AND "mangostana"[All Fields]) OR "garcinia mangostana"[All Fields] OR "mangosteen"[All Fields]) OR (("garcinia mangostana"[MeSH Terms] OR ("garcinia"[All Fields] AND "mangostana"[All Fields]) OR "garcinia mangostana"[All Fields]) AND ("extract"[All Fields] OR "extract s"[All Fields] OR "extractabilities"[All Fields] OR "extractability"[All Fields] OR "extractable"[All Fields] OR "extractables"[All Fields] OR "extractant"[All Fields] OR "extractants"[All Fields] OR "extracted"[All Fields] OR "extractibility"[All Fields] OR "extractible"[All Fields] OR "extracting"[All Fields] OR "extraction"[All Fields] OR "extractions"[All Fields] OR "extractive"[All Fields] OR "extractives"[All Fields] OR "extracts"[All Fields])) OR ("g"[All Fields] AND "mangostana"[All Fields] AND ("extract"[All Fields] OR "extract s"[All Fields] OR "extractabilities"[All Fields] OR "extractability"[All Fields] OR "extractable"[All Fields] OR "extractables"[All Fields] OR "extractant"[All Fields] OR "extractants"[All Fields] OR "extracted"[All Fields] OR "extractibility"[All Fields] OR "extractible"[All Fields] OR "extracting"[All Fields] OR "extraction"[All Fields] OR "extractions"[All Fields] OR "extractive"[All Fields] OR "extractives"[All Fields] OR "extracts"[All Fields])) OR (("garcinia mangostana"[MeSH Terms] OR ("garcinia"[All Fields] AND "mangostana"[All Fields]) OR "garcinia mangostana"[All Fields] OR "mangosteen"[All Fields]) AND ("extract"[All Fields] OR "extract s"[All Fields] OR "extractabilities"[All Fields] OR "extractability"[All Fields] OR "extractable"[All Fields] OR "extractables"[All Fields] OR "extractant"[All Fields] OR "extractants"[All Fields] OR "extracted"[All Fields] OR "extractibility"[All Fields] OR "extractible"[All Fields] OR "extracting"[All Fields] OR "extraction"[All Fields] OR "extractions"[All Fields] OR "extractive"[All Fields] OR "extractives"[All Fields] OR "extracts"[All Fields]))) AND ("diabetes mellitus"[MeSH Terms] OR ("diabetes"[All Fields] AND "mellitus"[All Fields]) OR "diabetes mellitus"[All Fields] OR ("glucose"[MeSH Terms] OR "glucose"[All Fields] OR "glucoses"[All Fields] OR "glucose s"[All Fields]) OR ("hyperglycaemia"[All Fields] OR "hyperglycemia"[MeSH Terms] OR "hyperglycemia"[All Fields] OR "hyperglycaemias"[All Fields] OR "hyperglycemias"[All Fields] OR "hyperglycemia s"[All Fields])) | 49 |

**Table S3.** Database search strategies from Embase database.

| **Step** | **Search Algorithm** | **Item founds** |
| --- | --- | --- |
| #1 | garcinia mangostana*':ti,ab,kw,de | 1023 |
| #2 | 'g. mangostana*':ti,ab,kw,de | 133 |
| #3 | 'garcinia mangostana'/exp | 867 |
| #4 | 'mangosteen*':ti,ab,kw,de | 734 |
| #5 | garcinia mangostana extract*':ti,ab,kw,de | 288 |
| #6 | garcinia mangostana extract'/exp | 277 |
| #7 | garcinia mangostana extract'/exp | 11 |
| #8 | 'mangosteen extract*':ti,ab,kw,de | 66 |
| #9 | #1 OR #2 OR #3 OR #4 OR #5 OR #6 OR #7 OR #8 | 1237 |
| #10 | 'diabetes mellitus*':ti,ab,kw,de | [1,186,624](https://www.embase.com/) |
| #11 | 'diabetes mellitus'/exp | 1,237,585 |
| #12 | 'glucose*':ti,ab,kw,de | 1,035,836 |
| #13 | 'glucose'/exp | 507,603 |
| #14 | 'hyperglycemia*':ti,ab,kw,de | 142,766 |
| #15 | 'hyperglycemia'/exp | 118,795 |
| #16 | #10 OR #11 OR #12 OR #13 OR #14 OR #15 | 1,972,343 |
| #17 | #9 AND #16 | 126 |

**Table S4.** Database search strategies from Scopus database.

| **Step** | **Search Algorithm** | **Item founds** |
| --- | --- | --- |
| #1 | TITLE-ABS-KEY ( garcinia AND mangostana ) | 1610 |
| #2 | TITLE-ABS-KEY ( g. AND mangostana ) | 380 |
| #3 | TITLE-ABS-KEY ( mangosteen ) | 1,553 |
| #4 | TITLE-ABS-KEY ( garcinia AND mangostana AND extract ) | 827 |
| #5 | TITLE-ABS-KEY ( g. AND mangostana AND extract ) | 223 |
| #6 | TITLE-ABS-KEY ( mangosteen AND extract ) | 621 |
| #7 | ( TITLE-ABS-KEY ( garcinia AND mangostana ) ) OR ( TITLE-ABS-KEY ( g. AND mangostana ) ) OR ( TITLE-ABS-KEY ( mangosteen ) ) OR ( TITLE-ABS-KEY ( garcinia AND mangostana AND extract ) ) OR ( TITLE-ABS-KEY ( g. AND mangostana AND extract ) ) OR ( TITLE-ABS-KEY ( mangosteen AND extract ) ) | 2,180 |
| #7 | TITLE-ABS-KEY ( diabetes AND mellitus ) | 918,775 |
| #8 | TITLE-ABS-KEY ( glucose ) | 1,047,261 |
| #10 | TITLE-ABS-KEY ( hyperglycemia ) | 130,202 |
| #11 | ( TITLE-ABS-KEY ( diabetes AND mellitus ) ) OR ( TITLE-ABS-KEY ( hyperglycemia ) ) OR ( TITLE-ABS-KEY ( glucose ) ) | 1,705,673 |
| #12 | ( TITLE-ABS-KEY ( garcinia AND mangostana ) ) OR ( ( TITLE-ABS-KEY ( g. AND mangostana ) ) OR ( TITLE-ABS-KEY ( mangosteen ) ) OR ( TITLE-ABS-KEY ( garcinia AND mangostana AND extract ) ) OR ( TITLE-ABS-KEY ( g. AND mangostana AND extract ) ) OR ( TITLE-ABS-KEY ( mangosteen AND extract ) ) ) AND ( TITLE-ABS-KEY ( diabetes AND mellitus ) ) ) OR ( TITLE-ABS-KEY ( glucose ) ) OR ( ( TITLE-ABS-KEY ( hyperglycemia ) ) | 131 |

**Table S5.**  League table showing the results of network meta-analysis comparing the effect of GM treatments in different concentrations or drug treatment on blood glucose level in diabetic rodent models at more than 2 weeks. GM and drug treatments are reported based on ranking. Number below of each treatment represent the mean difference (95% confidence interval) in blood glucose level (mg/dL) between row and the column.

| **A_GBC05** |  |  |  |  |  |  |  |
| --- | --- | --- | --- | --- | --- | --- | --- |
| -100.14  (-312.05,111.77) | **B_GME200** |  |  |  |  |  |  |
| -99.44  (-364.97,166.09) | 0.70  (-209.18,210.58) | **C_GME400** |  |  |  |  |  |
| -130.08  (-332.99,72.84) | -29.94  (-184.70,124.83) | -30.64  (-235.52,174.24) | **D_GME100** |  |  |  |  |
| -168.51  (-434.54,97.52) | -68.37  (-303.02,166.28) | -69.07  (-342.13,203.98) | -38.44  (-243.89,167.02) | **E_OLST100** |  |  |  |
| -176.12  (-388.96,36.71) | -75.98  (-256.64,104.68) | -76.68  (-311.44,158.08) | -46.05  (-201.72,109.63) | -7.61  (-218.24,203.01) | **F_GME50** |  |  |
| -279.24  (-600.18,41.69) | -179.11  (-471.94,113.73) | -179.81  (-501.90,142.29) | -149.17  (-431.18,132.85) | -110.73  (-433.16,211.70) | -103.12  (-396.42,190.17) | **G_GMM400** |  |
| **-316.50**  **(-519.59,-113.42)** | **-216.37**  **(-371.27,-61.47)** | **-217.07**  **(-421.98,-12.15)** | **-186.43**  **(-319.75,-53.11)** | -147.99  (-353.42,57.44) | -140.38  (-296.15,15.39) | -37.26  (-285.77,211.25) | **H_CTRL** |

*Note: GBC05, glibenclamide at 0.5 mg/kg body weight; GME50, ethanol extract of Garcinia mangostana at 50 mg/kg body weight; GME100, ethanol extract of Garcinia mangostana at 100 mg/kg body weight; GME200, ethanol extract of Garcinia mangostana at 200 mg/kg body weight; GME400, ethanol extract of Garcinia mangostana at 400 mg/kg body weight; OLST100, orlistat at 100 mg/kg body weight; GMM400, methanol extract of Garcinia mangostana at 400 mg/kg body weight; CTRL, diabetic control. Bold values indicate statistical significance.*

**Table S6.**  League table showing the results of network meta-analysis comparing the effect of GM treatments in different concentrations or drug treatment on total cholesterol in diabetic rodent models. GM and drug treatments are reported based on ranking. Number below of each treatment represent the mean difference (95% confidence interval) in total cholesterol (mg/dL) between row and the column. Treatments are arranged in order of the mean ranking from network meta-analysis of total cholesterol, for the best (left) to the worst (right).

| **A_GBC60** |  |  |  |  |  |  |  |  |  |
| --- | --- | --- | --- | --- | --- | --- | --- | --- | --- |
| **-44.49**  **(-66.29,-22.69)** | **B_GBC05** |  |  |  |  |  |  |  |  |
| **-47.16**  **(-69.31,-25.01)** | -2.67  (-13.21,7.87) | **C_GME200** |  |  |  |  |  |  |  |
| **-48.00**  **(-64.81,-31.19)** | -3.51  (-23.31,16.29) | -0.84  (-21.03,19.35) | **D_GM200** |  |  |  |  |  |  |
| **-64.12**  **(-84.80,-43.43)** | **-19.63**  **(-27.30,-11.96)** | **-16.96**  **(-25.57,-8.34)** | -16.12  (-34.69,2.45) | **E_GME100** |  |  |  |  |  |
| **-65.94**  **(-86.80,-45.09)** | **-21.45**  **(-29.26,-13.64)** | **-18.78**  **(-27.52,-10.05)** | -17.94  (-36.70,0.82) | -1.83  (-5.48,1.82) | **F_GME50** |  |  |  |  |
| **-67.55**  **(-90.94,-44.17)** | **-23.06**  **(-36.42,-9.71)** | **-20.39**  **(-34.31,-6.48)** | -19.55  (-41.10,1.99) | -3.44  (-14.40,7.52) | -1.61  (-13.12,9.90) | **G_OLST100** |  |  |  |
| **-93.00**  **(-108.25,-77.75)** | **-48.51**  **(-67.00,-30.02)** | **-45.84**  **(-64.74,-26.94)** | **-45.00**  **(-57.23,-32.77)** | **-28.88**  **(-46.05,-11.72)** | **-27.06**  **(-44.43,-9.68)** | **-25.45**  **(-45.79,-5.10)** | **H_GM100** |  |  |
| **-142.22**  **(-189.75,-94.69)** | **-97.73**  **(-141.60,-53.86)** | **-95.06**  **(-139.11,-51.01)** | **-94.22**  **(-140.87,-47.57)** | **-78.10**  **(-121.43,-34.77)** | **-76.28**  **(-119.69,-32.86)** | **-74.67**  **(-119.35,-29.98)** | **-49.22**  **(-95.33,-3.11)** | **J_GMM400** |  |
| **-108.00**  **(-128.12,-87.88)** | **-63.51**  **(-71.89,-55.13)** | **-60.84**  **(-70.09,-51.59)** | **-60.00**  **(-77.94,-42.06)** | **-43.88**  **(-48.68,-39.09)** | **-42.06**  **(-47.54,-36.57)** | **-40.45**  **(-52.37,-28.52)** | -15.00  (-31.49,1.49) | 34.22  (-8.84,77.28) | **I_CTRL** |

*Note: GBC05, glibenclamide at 0.5 mg/kg body weight; GBC60, glibenclamide at 60 mg/kg body weight; GM100, Garcinia mangostana at 100 mg/kg body weight; GM200, Garcinia mangostana at 200 mg/kg body weight; GME50, ethanol extract of Garcinia mangostana at 50 mg/kg body weight; GME100, ethanol extract of Garcinia mangostana at 100 mg/kg body weight; GME200, ethanol extract of Garcinia mangostana at 200 mg/kg body weight; GMM400, methanol extract of Garcinia mangostana at 400 mg/kg body weight; CTRL, diabetic control. Bold values indicate statistical significance.*

**Table S7.**  League table showing the results of network meta-analysis comparing the effect of GM treatments in different concentrations or drug treatment on triglyceride levels in diabetic rodent models. GM and drug treatments are reported based on ranking. Number below of each treatment represent the mean difference (95% confidence interval) in total cholesterol (mg/dL) between row and the column. Treatments are arranged in order of the mean ranking from network meta-analysis of triglyceride, for the best (left) to the worst (right).

| **A_GBC05** |  |  |  |  |  |  |  |  |  |
| --- | --- | --- | --- | --- | --- | --- | --- | --- | --- |
| -29.00  (-68.52,10.52) | **B_GME200** |  |  |  |  |  |  |  |  |
| -36.84  (-74.14,0.46) | -7.84  (-45.11,29.43) | **C_GME100** |  |  |  |  |  |  |  |
| **-45.44**  **(-82.77,-8.10)** | -16.44  (-53.75,20.87) | -8.59  (-40.21,23.02) | **D_GME50** |  |  |  |  |  |  |
| **-66.09**  **(-113.34,-18.85)** | -37.09  (-84.31,10.13) | -29.25  (-67.85,9.35) | -20.66  (-59.52,18.21) | **E_OLST100** |  |  |  |  |  |
| **-75.22**  **(-129.71,-20.74)** | -46.22  (-100.70,8.25) | -38.38  (-88.54,11.77) | -29.79  (-80.06,20.48) | -9.13  (-63.85,45.59) | **F_GBC60** |  |  |  |  |
| **-82.22**  **(-137.01,-27.44)** | -53.22  (-107.99,1.54) | -45.38  (-95.86,5.09) | -36.79  (-87.38,13.80) | -16.13  (-71.14,38.88) | -7.00  (-47.10,33.10) | **G_GM200** |  |  |  |
| **-90.22**  **(-144.27,-36.18)** | **-61.22**  **(-115.25,-7.20)** | **-53.38**  **(-103.05,-3.71)** | -44.79  (-94.57,5.00) | -24.13  (-78.40,30.14) | -15.00  (-54.08,24.08) | -8.00  (-47.49,31.49) | **H_GM100** |  |  |
| **-115.68**  **(-171.16,-60.21)** | **-86.68**  **(-142.14,-31.23)** | **-78.84**  **(-130.06,-27.62)** | **-70.25**  **(-121.58,-18.91)** | **-49.59**  **(-105.29,6.10)** | -40.46  (-96.96,16.04) | -33.46  (-90.24,23.32) | -25.46  (-81.52,30.60) | **I_GMM400** |  |
| **-116.22**  **(-154.00,-78.45)** | **-87.22**  **(-124.98,-49.47)** | **-79.38**  **(-110.59,-48.18)** | **-70.79**  **(-102.18,-39.40)** | **-50.13**  **(-88.24,-12.03)** | **-41.00**  **(-80.27,-1.73)** | -34.00  (-73.68,5.68) | -26.00  (-64.64,12.64) | -0.54  (-41.16,40.08) | **I_CTRL** |

*Note: GBC05, glibenclamide at 0.5 mg/kg body weight; GBC60, glibenclamide at 60 mg/kg body weight; GM100, Garcinia mangostana at 100 mg/kg body weight; GM200, Garcinia mangostana at 200 mg/kg body weight; GME50, ethanol extract of Garcinia mangostana at 50 mg/kg body weight; GME100, ethanol extract of Garcinia mangostana at 100 mg/kg body weight; GME200, ethanol extract of Garcinia mangostana at 200 mg/kg body weight; GMM400, methanol extract of Garcinia mangostana at 400 mg/kg body weight; CTRL, diabetic control. Bold values indicate statistical significance.*

**Table S8.** League table showing the results of network meta-analysis comparing the effect of GM treatments in different concentrations or drug treatment on LDL levels in diabetic rodent models. GM and drug treatments are reported based on ranking. Number below of each treatment represent the mean difference (95% confidence interval) in LDL (mg/dL) between row and the column. Treatments are arranged in order of the mean ranking from network meta-analysis of triglyceride, for the best (left) to the worst (right).

| **A_GBC60** |  |  |  |  |  |  |  |  |  |
| --- | --- | --- | --- | --- | --- | --- | --- | --- | --- |
| **-52.00**  **(-76.63,-27.37)** | **B_GM200** |  |  |  |  |  |  |  |  |
| **-55.93**  **(-88.19,-23.68)** | -3.93  (-34.79,26.92) | **C_GBC05** |  |  |  |  |  |  |  |
| **-57.55**  **(-89.98,-25.13)** | -5.55  (-36.58,25.47) | -1.62  (-22.11,18.87) | **D_GME200** |  |  |  |  |  |  |
| **-73.07**  **(-102.84,-43.30)** | -21.07  (-49.32,7.17) | -17.14  (-35.29,1.02) | -15.52  (-33.97,2.94) | **E_GME50** |  |  |  |  |  |
| **-73.39**  **(-106.45,-40.34)** | -21.39  (-53.08,10.29) | -17.46  (-41.88,6.96) | -15.84  (-40.48,8.81) | -0.32  (-19.89,19.25) | **F_OLST100** |  |  |  |  |
| **-73.75**  **(-103.35,-44.15)** | -21.75  (-49.81,6.31) | **-17.82**  **(-35.53,-0.10)** | -16.20  (-34.22,1.83) | -0.68  (-13.53,12.17) | -0.36  (-19.74,19.02) | **G_GME100** |  |  |  |
| **-98.00**  **(-121.08,-74.92)** | **-46.00**  **(-67.07,-24.93)** | **-42.07**  **(-71.69,-12.44)** | **-40.45**  **(-70.25,-10.64)** | -24.93  (-51.83,1.97) | -24.61  (-55.10,5.88) | -24.25  (-50.95,2.46) | **H_GM100** |  |  |
| **-140.89**  **(-190.90,-90.88)** | **-88.89**  **(-138.01,-39.77)** | **-84.96**  **(-131.33,-38.58)** | **-83.34**  **(-129.83,-36.84)** | **-67.82**  **(-112.50,-23.14)** | **-67.50**  **(-114.43,-20.57)** | **-67.14**  **(-111.70,-22.57)** | -42.89  (-91.24,5.46) | **J_GMM400** |  |
| **-110.00**  **(-136.37,-83.63)** | **-58.00**  **(-82.63,-33.37)** | **-54.07**  **(-72.64,-35.49)** | **-52.45**  **(-71.31,-33.58)** | **-36.93**  **(-50.75,-23.11)** | **-36.61**  **(-56.54,-16.68)** | **-36.25**  **(-49.69,-22.81)** | -12.00  (-35.08,11.08) | 30.89  (-11.60,73.38) | **I_CTRL** |

*Note: GBC05, glibenclamide at 0.5 mg/kg body weight; GBC60, glibenclamide at 60 mg/kg body weight; GM100, Garcinia mangostana at 100 mg/kg body weight; GM200, Garcinia mangostana at 200 mg/kg body weight; GME50, ethanol extract of Garcinia mangostana at 50 mg/kg body weight; GME100, ethanol extract of Garcinia mangostana at 100 mg/kg body weight; GME200, ethanol extract of Garcinia mangostana at 200 mg/kg body weight; GMM400, methanol extract of Garcinia mangostana at 400 mg/kg body weight; CTRL, diabetic control. Bold values indicate statistical significance.*

**Table S9.**  League table showing the results of network meta-analysis comparing the effect of GM treatments in different concentrations or drug treatment on HDL-C levels in diabetic rodent models. GM and drug treatments are reported based on ranking. Number below of each treatment represent the mean difference (95% confidence interval) in HDL-C (mg/dL) between row and the column. Treatments are arranged in order of the mean ranking from network meta-analysis of triglyceride, for the best (left) to the worst (right).

| **A_GBC60** |  |  |  |  |  |  |  |  |  |
| --- | --- | --- | --- | --- | --- | --- | --- | --- | --- |
| 5.74  (-11.80,23.28) | **B_GBC05** |  |  |  |  |  |  |  |  |
| 6.00  (-6.74,18.74) | 0.26  (-17.23,17.75) | **C_GM200** |  |  |  |  |  |  |  |
| 7.37  (-17.55,32.29) | 1.63  (-22.95,26.21) | 1.37  (-23.51,26.25) | **D_GMM400** |  |  |  |  |  |  |
| 9.00  (-3.74,21.74) | 3.26  (-14.23,20.75) | 3.00  (-9.66,15.66) | 1.63  (-23.25,26.51) | **E_GM100** |  |  |  |  |  |
| 10.50  (-7.03,28.04) | 4.76  (-8.20,17.72) | 4.50  (-12.98,21.98) | 3.13  (-21.44,27.70) | 1.50  (-15.98,18.98) | **F_GME200** |  |  |  |  |
| 14.17  (-2.12,30.47) | 8.43  (-3.68,20.54) | 8.17  (-8.06,24.41) | 6.80  (-16.90,30.51) | 5.17  (-11.06,21.41) | 3.67  (-8.42,15.77) | **H_GME50** |  |  |  |
| 14.96  (-1.08,31.00) | 9.22  (-2.73,21.18) | 8.96  (-7.02,24.94) | 7.59  (-15.93,31.12) | 5.96  (-10.02,21.94) | 4.46  (-7.48,16.41) | 0.79  (-9.13,10.70) | **I_GME100** |  |  |
| 19.45  (-0.13,39.02) | 13.71  (-3.68,31.09) | 13.45  (-6.08,32.97) | 12.08  (-13.99,38.14) | 10.45  (-9.08,29.97) | 8.95  (-8.44,26.33) | 5.27  (-9.92,20.46) | 4.48  (-10.29,19.26) | **J_OLST100** |  |
| 11.00  (-1.74,23.74) | 5.26  (-6.80,17.32) | 5.00  (-7.66,17.66) | 3.63  (-17.78,25.04) | 2.00  (-10.66,14.66) | 0.50  (-11.55,12.55) | -3.17  (-13.34,6.99) | -3.96  (-13.71,5.78) | -8.45  (-23.31,6.42) | **G_CTRL** |

*Note: GBC05, glibenclamide at 0.5 mg/kg body weight; GBC60, glibenclamide at 60 mg/kg body weight; GM100, Garcinia mangostana at 100 mg/kg body weight; GM200, Garcinia mangostana at 200 mg/kg body weight; GME50, ethanol extract of Garcinia mangostana at 50 mg/kg body weight; GME100, ethanol extract of Garcinia mangostana at 100 mg/kg body weight; GME200, ethanol extract of Garcinia mangostana at 200 mg/kg body weight; GMM400, methanol extract of Garcinia mangostana at 400 mg/kg body weight; CTRL, diabetic control. Bold values indicate statistical significance.*

**Table S10**. Sensitivity analyses according to the blood glucose measured within 2 weeks.

| Treatment | Primary analysis  (main findings) | SURCA rank | Excluded studies with missing SD values | SURCA rank |
| --- | --- | --- | --- | --- |
| Number of studies | 6 |  | 5 |  |
| GBC60 | -127.46 (-140.22, -114.69) | 1.9 | -126.18 (-139.13, (-113.242) | 1.9 |
| GBC05 | -131.49 (-197.38, -65.61) | 2.8 | -131.49 (-197.37, -65.61) | 2.7 |
| GM200 | -116.25 (-139.52, -92.98) | 3.6 | -114.31 (-137.80, -90.81) | 3.7 |
| GMA200 | -115.22 (134.46, -95.97) | 3.9 | -114.48 (-133.77, -95.20) | 3.6 |
| GM100 | -108.12 (-135.59, 92.98) | 4.9 | -101.35 (-131.03, -71.66) | 5.7 |
| GMA100 | -101.90 (-129.48, -74.32) | 5.8 | -101.16 (-128.77, -73.55) | 5.7 |
| GME200 | -86.41 (-121.42, -51.41) | 7.4 | -86.41 (-121.42, -51.41) | 7.2 |
| GME50 | -82.16 (-107.17, -57.16) | 8.0 | -82.16 (-107.17, -57.16) | 7.9 |
| GMH18 | -80.45 (-109.20, -51.71) | 8.4 | -80.45 (-109.20, -51.71) | 8.3 |
| GME100 | -75.62 (-105.37, -45.88) | 9.3 | -75.62 (-105.37, -45.88) | 9.2 |
| MTF100 | -70.01 (-95.90, -44.12) | 10.4 | -70.01 (-95.90, -44.12) | 10.4 |
| GMC80 | -41.21 (-89.94, 7.51) | 11.7 | -41.21 (-89.94, 7.51) | 11.7 |
| CTRL | Reference | 13.0 | Reference | 12.9 |

**Table S11.** CINeMa framework confidence ratings for each available pairwise comparison.

| Comparison | Number of studies | Within-study bias | Reporting bias | Indirectness | Imprecision | Heterogeneity | Incoherence | Confidence rating |
| --- | --- | --- | --- | --- | --- | --- | --- | --- |
| CTRL:GBC05 | 1 | No concerns | Low risk | No concerns | No concerns | No concerns | No concerns | High |
| CTRL:GBC60 | 2 | No concerns | Low risk | No concerns | No concerns | No concerns | No concerns | High |
| CTRL:GM100 | 2 | No concerns | Low risk | No concerns | No concerns | No concerns | No concerns | High |
| CTRL:GM200 | 1 | No concerns | Low risk | No concerns | No concerns | No concerns | No concerns | High |
| CTRL:GMA100 | 1 | No concerns | Low risk | No concerns | No concerns | No concerns | No concerns | High |
| CTRL:GMA200 | 1 | No concerns | Low risk | No concerns | No concerns | No concerns | No concerns | High |
| CTRL:GMC80 | 1 | No concerns | Low risk | No concerns | Some concerns | No concerns | No concerns | High |
| CTRL:GME100 | 2 | No concerns | Low risk | No concerns | No concerns | No concerns | No concerns | High |
| CTRL:GME200 | 2 | No concerns | Low risk | No concerns | No concerns | No concerns | No concerns | High |
| CTRL:GME50 | 3 | No concerns | Low risk | No concerns | No concerns | No concerns | No concerns | High |
| CTRL:GMH18 | 1 | No concerns | Low risk | No concerns | No concerns | No concerns | No concerns | High |
| CTRL:MTF100 | 2 | No concerns | Low risk | No concerns | No concerns | No concerns | No concerns | High |
| GBC05:GME100 | 1 | No concerns | Low risk | No concerns | Some concerns | No concerns | No concerns | High |
| GBC05:GME200 | 1 | No concerns | Low risk | No concerns | Some concerns | Some concerns | No concerns | Moderate |
| GBC05:GME50 | 1 | No concerns | Low risk | No concerns | Some concerns | No concerns | No concerns | High |
| GBC60:GM100 | 1 | No concerns | Low risk | No concerns | Some concerns | No concerns | No concerns | High |
| GBC60:GM200 | 1 | No concerns | Low risk | No concerns | No concerns | Some concerns | No concerns | High |
| GBC60:GMA100 | 1 | No concerns | Low risk | No concerns | Some concerns | No concerns | No concerns | High |
| GBC60:GMA200 | 1 | No concerns | Low risk | No concerns | No concerns | Some concerns | No concerns | High |
| GM100:GM200 | 1 | No concerns | Low risk | No concerns | Some concerns | No concerns | No concerns | High |
| GMA100:GMA200 | 1 | No concerns | Low risk | No concerns | Some concerns | No concerns | No concerns | High |
| GMC80:GME50 | 1 | No concerns | Low risk | No concerns | Some concerns | No concerns | No concerns | High |
| GMC80:GMH18 | 1 | No concerns | Low risk | No concerns | Some concerns | No concerns | No concerns | High |
| GMC80:MTF100 | 1 | No concerns | Low risk | No concerns | Some concerns | No concerns | No concerns | High |
| GME100:GME200 | 2 | No concerns | Low risk | No concerns | Some concerns | No concerns | No concerns | High |
| GME100:GME50 | 2 | No concerns | Low risk | No concerns | No concerns | No concerns | No concerns | High |
| GME100:MTF100 | 1 | No concerns | Low risk | No concerns | No concerns | No concerns | No concerns | High |
| GME200:GME50 | 2 | No concerns | Low risk | No concerns | No concerns | Some concerns | No concerns | High |
| GME200:MTF100 | 1 | No concerns | Low risk | No concerns | Some concerns | No concerns | No concerns | High |
| GME50:GMH18 | 1 | No concerns | Low risk | No concerns | No concerns | No concerns | No concerns | High |
| GME50:MTF100 | 2 | No concerns | Low risk | No concerns | No concerns | No concerns | No concerns | High |
| GMH18:MTF100 | 1 | No concerns | Low risk | No concerns | No concerns | No concerns | No concerns | High |
| GBC05:GBC60 | 0 | No concerns | Low risk | No concerns | Major concerns | No concerns | No concerns | Moderate |
| GBC05:GM100 | 0 | No concerns | Low risk | No concerns | Major concerns | No concerns | No concerns | Moderate |
| GBC05:GM200 | 0 | No concerns | Low risk | No concerns | Major concerns | No concerns | No concerns | Moderate |
| GBC05:GMA100 | 0 | No concerns | Low risk | No concerns | Major concerns | No concerns | No concerns | Moderate |
| GBC05:GMA200 | 0 | No concerns | Low risk | No concerns | Major concerns | No concerns | No concerns | Moderate |
| GBC05:GMC80 | 0 | No concerns | Low risk | No concerns | No concerns | Some concerns | No concerns | High |
| GBC05:GMH18 | 0 | No concerns | Low risk | No concerns | Some concerns | No concerns | No concerns | High |
| GBC05:MTF100 | 0 | No concerns | Low risk | No concerns | Some concerns | No concerns | No concerns | High |
| GBC60:GMC80 | 0 | No concerns | Low risk | No concerns | No concerns | No concerns | No concerns | High |
| GBC60:GME100 | 0 | No concerns | Low risk | No concerns | No concerns | No concerns | No concerns | High |
| GBC60:GME200 | 0 | No concerns | Low risk | No concerns | No concerns | Some concerns | No concerns | High |
| GBC60:GME50 | 0 | No concerns | Low risk | No concerns | No concerns | No concerns | No concerns | High |
| GBC60:GMH18 | 0 | No concerns | Low risk | No concerns | No concerns | No concerns | No concerns | High |
| GBC60:MTF100 | 0 | No concerns | Low risk | No concerns | No concerns | No concerns | No concerns | High |
| GM100:GMA100 | 0 | No concerns | Low risk | No concerns | Some concerns | Some concerns | No concerns | Moderate |
| GM100:GMA200 | 0 | No concerns | Low risk | No concerns | Some concerns | No concerns | No concerns | High |
| GM100:GMC80 | 0 | No concerns | Low risk | No concerns | No concerns | Some concerns | No concerns | High |
| GM100:GME100 | 0 | No concerns | Low risk | No concerns | Some concerns | No concerns | No concerns | High |
| GM100:GME200 | 0 | No concerns | Low risk | No concerns | Some concerns | No concerns | No concerns | High |
| GM100:GME50 | 0 | No concerns | Low risk | No concerns | Some concerns | No concerns | No concerns | High |
| GM100:GMH18 | 0 | No concerns | Low risk | No concerns | Some concerns | No concerns | No concerns | High |
| GM100:MTF100 | 0 | No concerns | Low risk | No concerns | No concerns | Some concerns | No concerns | High |
| GM200:GMA100 | 0 | No concerns | Low risk | No concerns | Some concerns | No concerns | No concerns | High |
| GM200:GMA200 | 0 | No concerns | Low risk | No concerns | No concerns | No concerns | No concerns | High |
| GM200:GMC80 | 0 | No concerns | Low risk | No concerns | No concerns | No concerns | No concerns | High |
| GM200:GME100 | 0 | No concerns | Low risk | No concerns | No concerns | Some concerns | No concerns | High |
| GM200:GME200 | 0 | No concerns | Low risk | No concerns | Some concerns | No concerns | No concerns | High |
| GM200:GME50 | 0 | No concerns | Low risk | No concerns | Some concerns | No concerns | No concerns | High |
| GM200:GMH18 | 0 | No concerns | Low risk | No concerns | Some concerns | No concerns | No concerns | High |
| GM200:MTF100 | 0 | No concerns | Low risk | No concerns | No concerns | No concerns | No concerns | High |
| GMA100:GMC80 | 0 | No concerns | Low risk | No concerns | No concerns | Some concerns | No concerns | High |
| GMA100:GME100 | 0 | No concerns | Low risk | No concerns | Some concerns | No concerns | No concerns | High |
| GMA100:GME200 | 0 | No concerns | Low risk | No concerns | Some concerns | Some concerns | No concerns | Moderate |
| GMA100:GME50 | 0 | No concerns | Low risk | No concerns | Some concerns | No concerns | No concerns | High |
| GMA100:GMH18 | 0 | No concerns | Low risk | No concerns | Some concerns | No concerns | No concerns | High |
| GMA100:MTF100 | 0 | No concerns | Low risk | No concerns | Some concerns | No concerns | No concerns | High |
| GMA200:GMC80 | 0 | No concerns | Low risk | No concerns | No concerns | No concerns | No concerns | High |
| GMA200:GME100 | 0 | No concerns | Low risk | No concerns | No concerns | Some concerns | No concerns | High |
| GMA200:GME200 | 0 | No concerns | Low risk | No concerns | Some concerns | No concerns | No concerns | High |
| GMA200:GME50 | 0 | No concerns | Low risk | No concerns | No concerns | Some concerns | No concerns | High |
| GMA200:GMH18 | 0 | No concerns | Low risk | No concerns | No concerns | Some concerns | No concerns | High |
| GMA200:MTF100 | 0 | No concerns | Low risk | No concerns | No concerns | No concerns | No concerns | High |
| GMC80:GME100 | 0 | No concerns | Low risk | No concerns | Some concerns | No concerns | No concerns | High |
| GMC80:GME200 | 0 | No concerns | Low risk | No concerns | Some concerns | No concerns | No concerns | High |
| GME100:GMH18 | 0 | No concerns | Low risk | No concerns | No concerns | Some concerns | No concerns | High |
| GME200:GMH18 | 0 | No concerns | Low risk | No concerns | Some concerns | No concerns | No concerns | High |

**Note:** GBC05, glibenclamide at 0.5 mg/kg body weight; GBC60, glibenclamide at 60 mg/kg body weight; GM100, Garcinia mangostana extract at 100 mg/kg bodyweight; GM200, Garcinia mangostana extract at 200 mg/kg bodyweight; GMA100, aqueous extract of Garcinia mangostana at 100 mg/kg body weight; GMA200, aqueous extract of Garcinia mangostana at 200 mg/kg body weight; GMC80, chloroform extract of Garcinia mangostana at 80 mg/kg body weight; GME50, ethanol extract of Garcinia mangostana at 50 mg/kg body weight; GME100, ethanol extract of Garcinia mangostana at 100 mg/kg body weight; GME200, ethanol extract of Garcinia mangostana at 200 mg/kg body weight; GMH18, hexane extract of Garcinia mangostana at 18 mg/kg body weight; MTF100, metformin at 100 mg/kg body weight; CTRL, diabetic control.


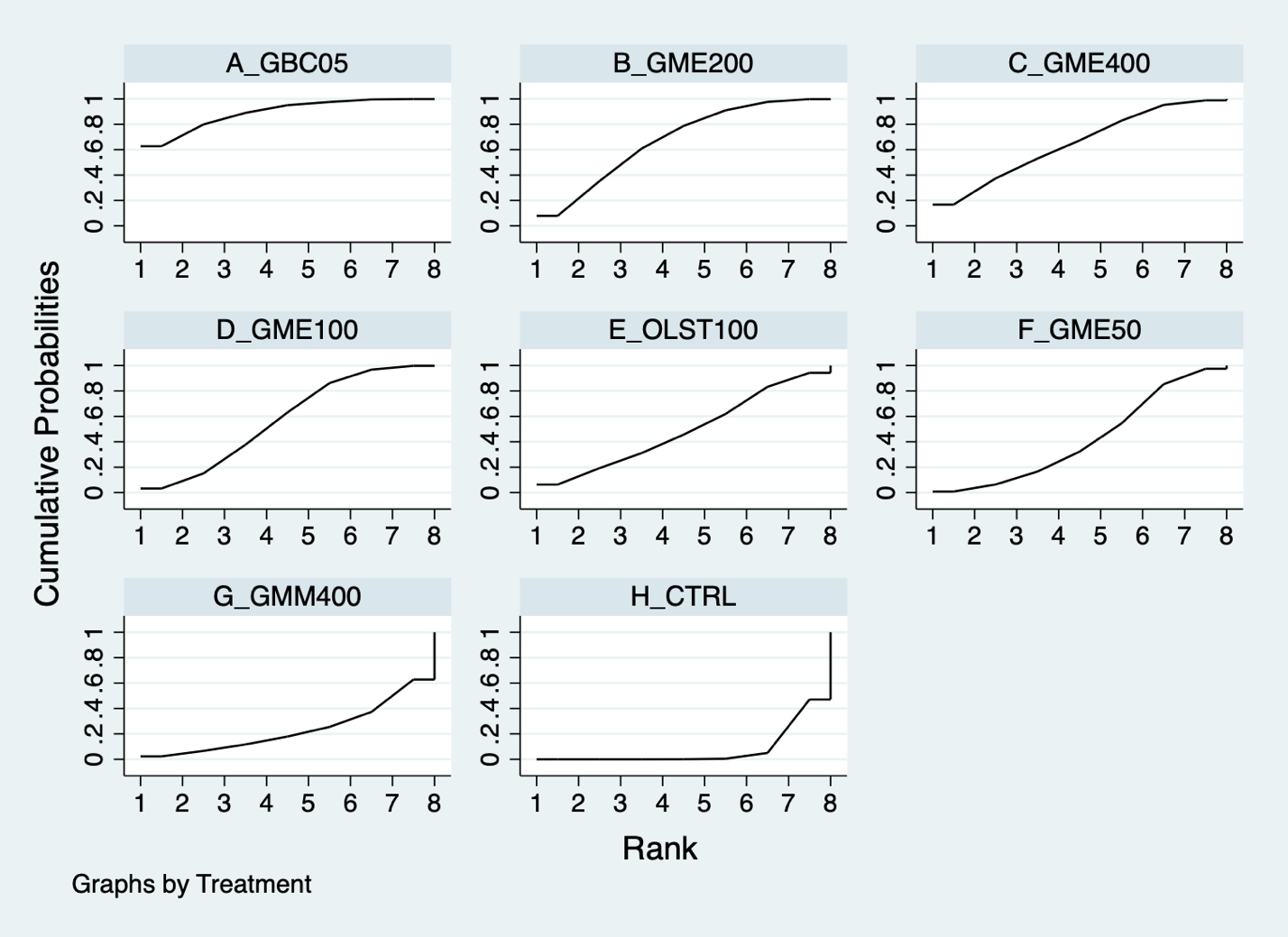


**Figure S1.** The surface under the cumulative ranking curve (SURCA) of each GM or drug treatment for blood glucose improvement at more than 2 weeks.


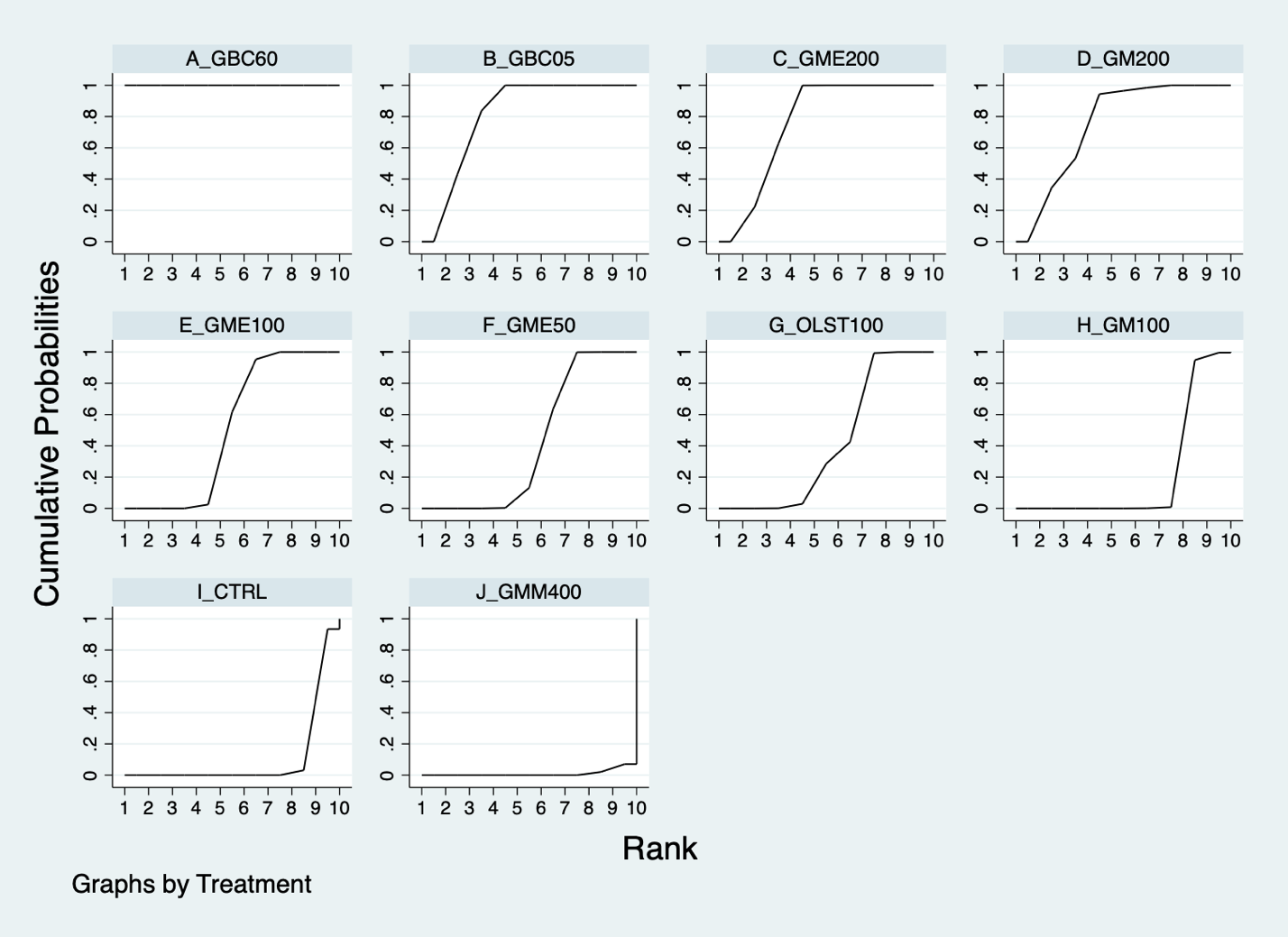


**Figure S2.** The surface under the cumulative ranking curve (SURCA) of each GM or drug treatment for total cholesterol improvement.


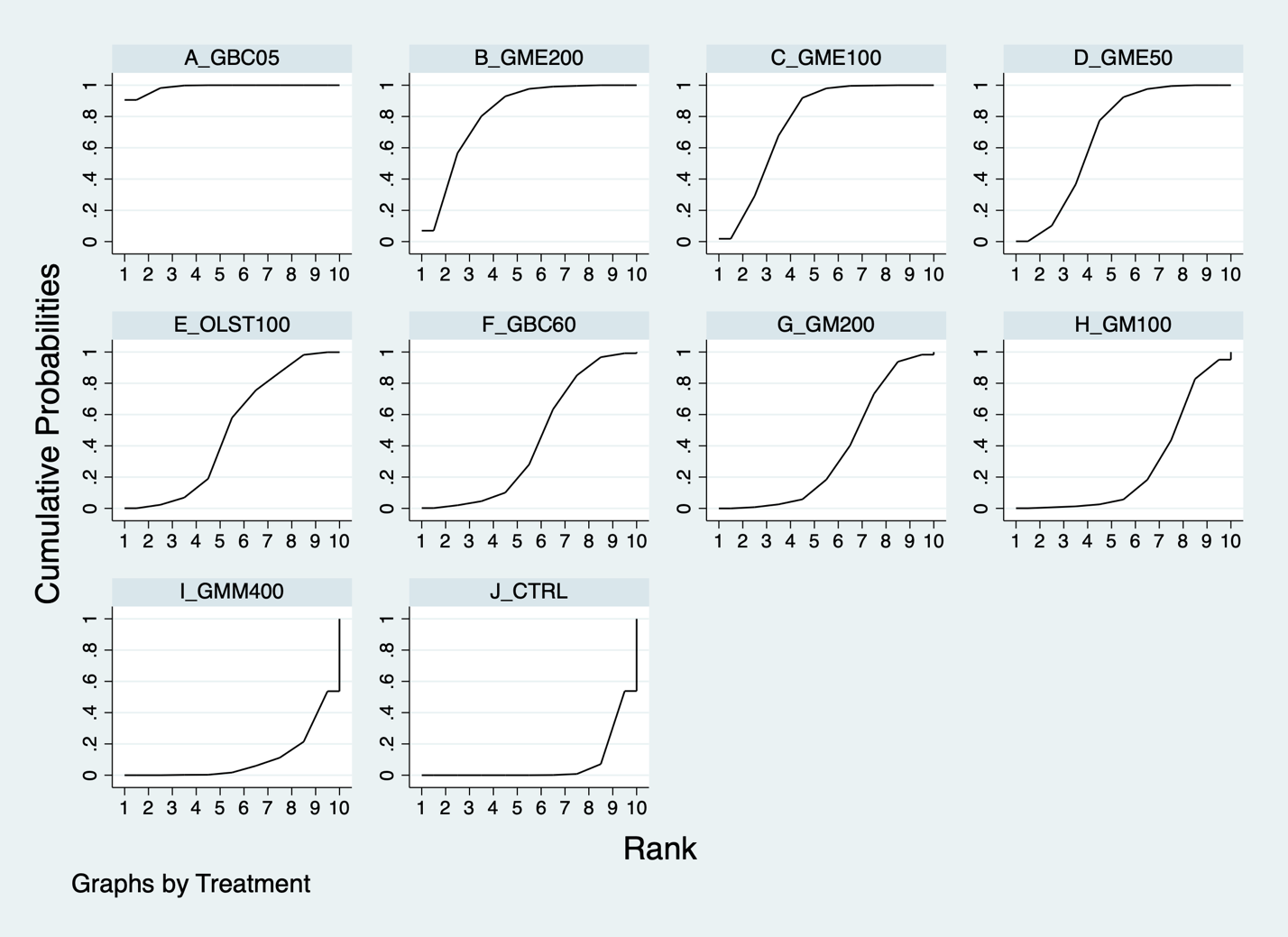


**Figure S3**. The surface under the cumulative ranking curve (SURCA) of each GM or drug treatment for triglyceride improvement.


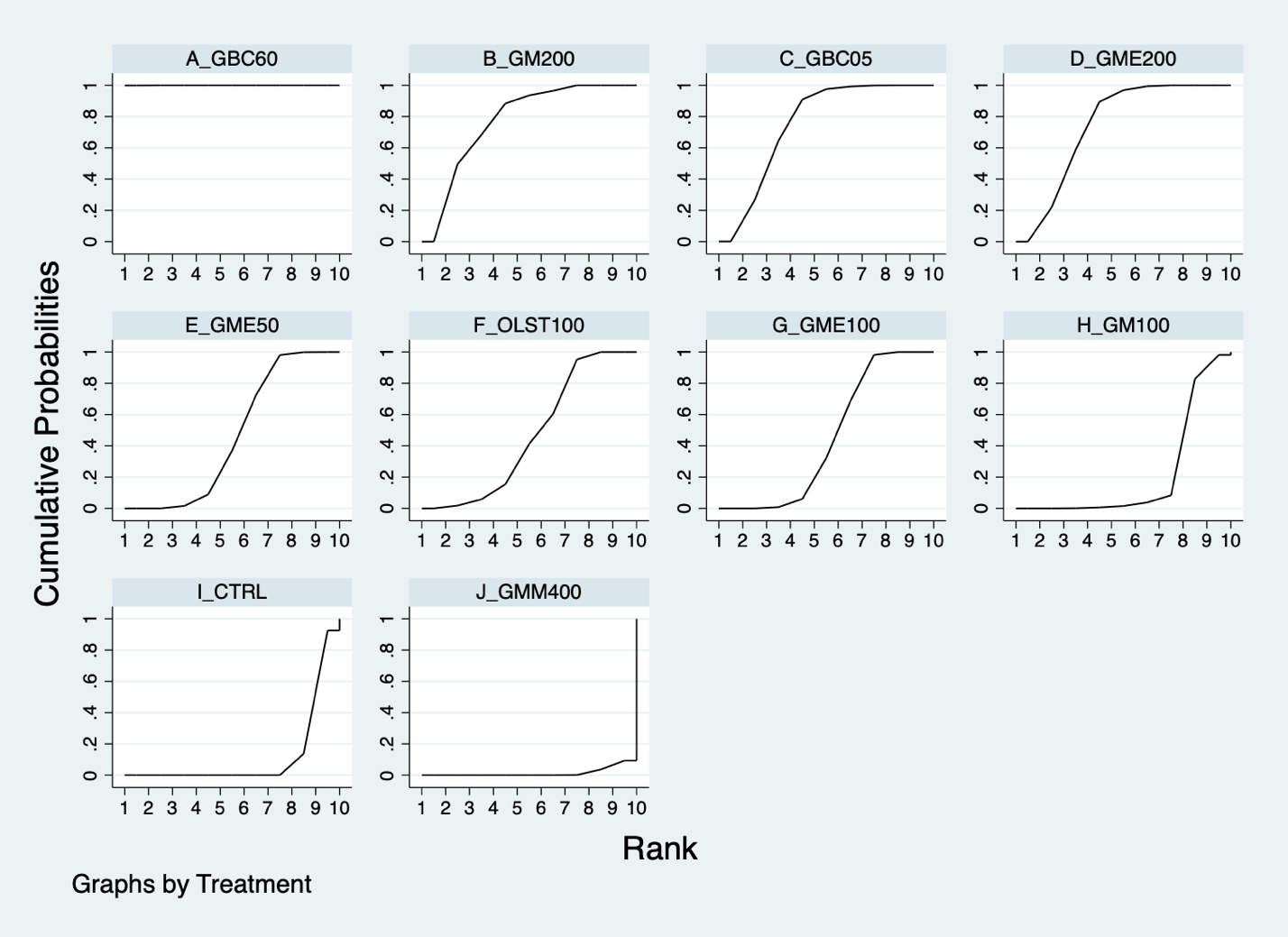


**Figure S4.** The surface under the cumulative ranking curve (SURCA) of each GM or drug treatment for LDL-C improvement.


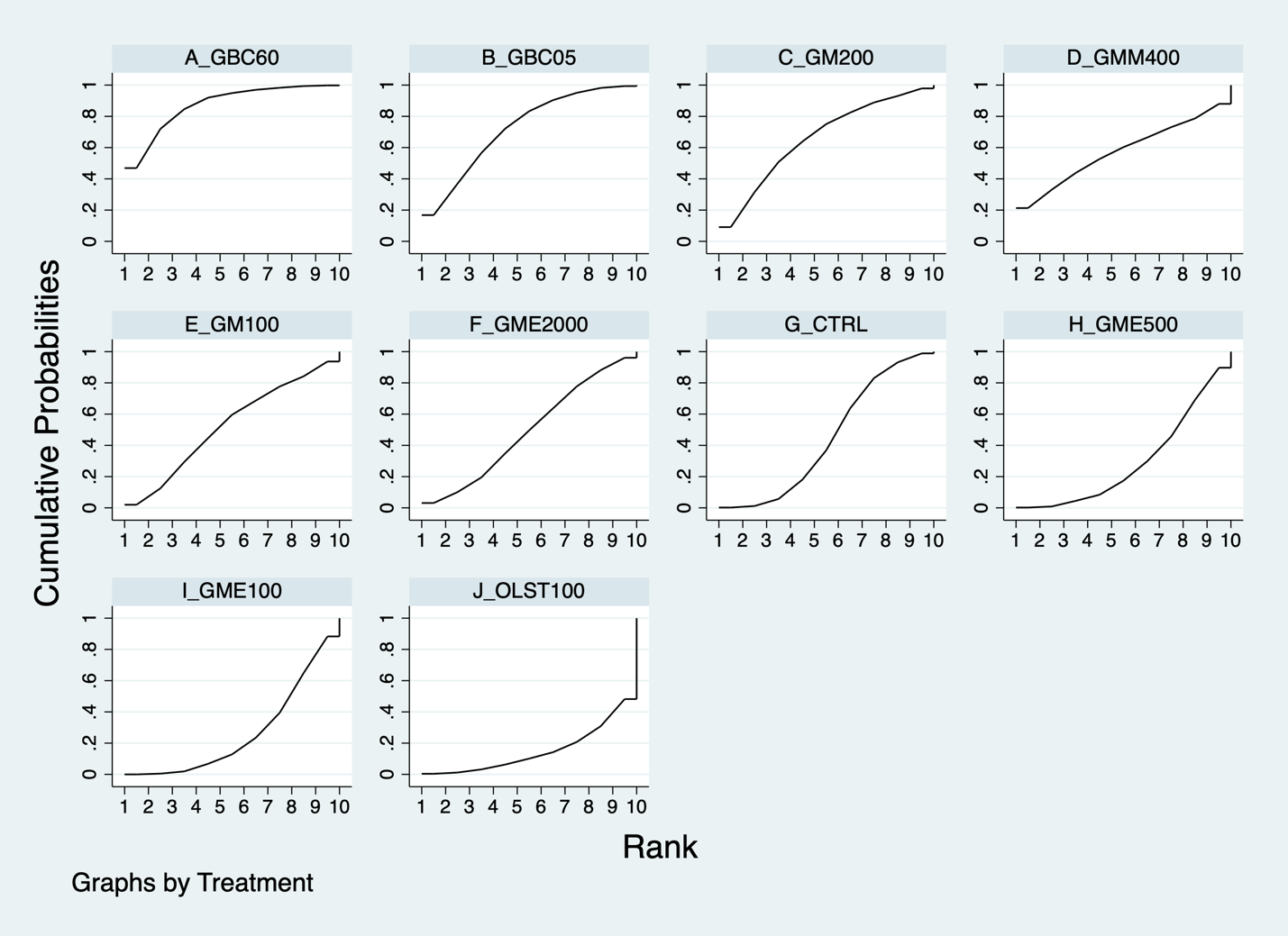


**Figure S5.** The surface under the cumulative ranking curve (SURCA) of each GM or drug treatment for HDL-C improvement.
